# Association of Bisphenol Exposure and Serum Hypothalamic–Pituitary–Thyroid Axis Hormone Levels in Adults and Pregnant Women: A Systematic Review and Meta-Analysis

**DOI:** 10.3390/toxics13100836

**Published:** 2025-09-30

**Authors:** Mazhar Sultan, Xuan Ma, Qiurun Yu, Francis Manyori Bigambo, Yufeng Tang, Natasha Chitakwa, Farah Kafauit, Qinrou Chen, Quanquan Guan, Yankai Xia

**Affiliations:** 1 State Key Laboratory of Reproductive Medicine and Offspring Health, Center for Global Health, School of Public Health, Nanjing Medical University, Nanjing 211166, China; mazharsa03@njmu.edu.cn (M.S.);; 2 Key Laboratory of Modern Toxicology of Ministry of Education, School of Public Health, Nanjing Medical University, Nanjing 211166, China; 3 The Affiliated Wuxi Center for Disease Control and Prevention of Nanjing Medical University, Wuxi Center for Disease Control and Prevention, Wuxi Medical Center, Nanjing Medical University, Nanjing 210000, China; maxuan@njmu.edu.cn; 4 Children’s Hospital of Nanjing Medical University, Nanjing 211166, China; 5 Department of Policy and Public Management, Zanvyl Krieger School of Arts and Sciences, Johns Hopkins University, Washington, DC 20001, USA; 6 Department of Clinical Pharmacology, Sir Run Run Hospital, Nanjing Medical University, Nanjing 211166, China

**Keywords:** bisphenols, thyroid hormones, adults, pregnant woman, meta-analysis

## Abstract

Background: Bisphenols (BPs) are present in medical instruments, plastic containers, and personal care products (PCPs). Bisphenol A has been replaced by its alternatives, bisphenol S, F, AF, and B. Due to the awareness of their toxicity, mixed exposure to these alternatives at the regional level has been given less attention; there is a need to study this area of research. This meta-analysis examined the exposure of urinary bisphenol A and its metabolites to blood Hypothalamic–Pituitary–Thyroid axis hormones (HPT axis hormones) in pregnant women and adult males and females. We searched Embase, PubMed, Web of Science, Cochrane Library, and CINAHL until 8 January 2025, yielding 4588 articles using the PECO framework. Quality assessment was done using AHRQ: Agency for Healthcare Research and Quality for cross-sectional and NOS: Newcastle Ottawa Scale for cohort studies, with combined exposure evaluated using random and fixed-effect models. The *I*^2^ test assessed heterogeneity. We included eighteen studies for the final analysis. Fixed-effect model estimates revealed that BPA is negatively associated with thyroid-stimulating hormone (TSH) in female and male adults (β = −0.02; 95% CI = −0.04 to −0.01); (β = −0.08; 95% CI = −0.14 to −0.02). In Females, BPA was positively associated with free thyroxine, FT4 (β = 0.001, 95% CI, 0.001 to 0.001). In the male group, BPA was negatively associated with FT4 (β = −0.001, 95% CI, −0.001 to −0.001). As per pregnant women, there was no association found between exposure to bisphenols and total Thyroxine (TT4), FT4, and TSH in both trimesters (β = 0.010, 95% CI = −0.030 to 0.050); (β = 0.001, 95% CI = −0.010 to 0.010); (β = −0.001, 95% CI = −0.010 to 0.001), respectively, for early pregnancy. Bisphenols can significantly influence HPT axis hormones in adult males, females, and pregnant women. Gender-based studies were observed, concluding that adult females are more affected by bisphenol exposures than adult males. The subgroup analysis based on the regions did not reveal any associations.

## 1. Introduction

Thyroid function and the activity of thyroid hormones are tightly controlled during fetal development and maturation, and they have a crucial impact on growth and metabolism. Humans are regularly exposed to endocrine-disrupting chemicals (EDCs) [1]. EDCs influence the thyroid functions, liver, heart, and reproductive systems through inhalation, ingestion, skin/eye contact, or maternofetal transfer [2]. Recently, it has been highlighted that chemicals like bisphenol A, triclosan (TCS), and many others disrupt the endocrine system. These chemicals are mostly found in water bodies, sediments, and aquatic species [3,4]. They can interfere with human hormone activity, leading to hormone problems and disorders. Bisphenol A and its other alternatives, often migrating from plastic boxes, are found in PCPs, including soaps and toothpaste [5]. In a recent study, bisphenol S (BPS) was detected in personal care product samples, with the highest detection frequency (71.5%) among bisphenols. This was followed by BPAF (70.8%), BPAP (63.0%), BPA (57.7%), BPF (56.7%), BPB (48.2%), BPP (39.7%), and BPZ (18.0%) [6]. Another study revealed that BPS is more often detected in PCPs, while in lotion and mask products, BPF and BPA are abundant [7]. Bisphenols are an often-used class of environmental endocrine disruptors used for polycarbonate plastics and epoxy resins, including BPB, BPA, BPS, tetrabromobisphenol A (TBBPA), and bisphenol F (BPF); these substances are widely detected in the human body and may disrupt thyroid hormone homeostasis [8]. Based on more than 46,000 urine measurements [9], it was reported that the global average daily BPA consumption is 30.76 ng/kg bw/day, and the maximum daily exposure for adults is 11 ng/kg bw/day.

There is abundant evidence from populations, animals, and in vitro studies indicating that BPA exposure can affect thyroid hormones. The widespread usage and manufacture of BPA raise health concerns. BPA can affect thyroid function by disrupting cellular communication and transcription [10]. Another study showed BPA’s negative effects on amphibian metabolism in Xenopus laevis. BPA binds to thyroid hormone receptor Alpha and thyroid hormone receptor Beta, opposing Triiodothyronine (T3), as suggested by the in vitro studies [11].

Epidemiological studies examined how prenatal BPA exposure affected pregnant mothers and their newborns’ thyroid axes [12,13,14]. Several research findings proved the adverse effect of BPA on human Hypothalamic–Pituitary–Thyroid (HPT) axis hormones [15]. Following regulatory restrictions on the use of BPA, structurally related analogs such as bisphenol S (BPS) and bisphenol F (BPF) have been increasingly adopted as substitutes. However, these alternatives cannot be regarded as safe replacements, since accumulating evidence indicates comparable endocrine-disrupting potential [16,17]. There is still insufficient attention to some alternatives to BPA, and research results are inconsistent.

The physiological functions of the thyroid axis vary in different populations. Cardiovascular and diabetes diseases can be caused by TH level changes [18,19]. BPs can cause hypothyroidism, which impacts fertility, pregnancy, and child health [20]. Hypothyroidism during pregnancy increases the risk of early delivery and miscarriage [21]. A study showed that BPA and its current alternatives are harmful to pregnant women [22]. Another study found that bisphenols’ developmental toxicity during pregnancy could affect offspring HPT axis hormones (FT3, TSH, T3, T4, and FT4) in a sex-specific manner. Several phthalates and BPA exposures changed circulatory HPT axis hormone levels in Korean adults [23]. BPA affects Chinese adults’ HPT axis hormones, more in women than men [24]. The US population similarly showed an inverse connection between urine BPA and total T4 and TSH [25]. The widespread presence of BPA and its structural analogues in PCPs needs more research to address more health issues. To our knowledge, no meta-analysis has examined the association of BPs with seven HPT axis hormones. Most of the current research is limited to specific population trends.

We evaluated the link between bisphenol A and its seven metabolites and HPT axis hormone levels in the serum of pregnant women, adult males, and females across the population, regions, and various bisphenol exposures.

## 2. Materials and Methods

### 2.1. Literature Search, Inclusion Criteria, and Data Extraction

We searched PubMed, Embase, Web of Science, CINAHL, and Cochrane Library for relevant literature from inception through 8 January 2025. The search strategy is the following:

(((((((((((Bisphenol A) OR (BPA)) OR (Bisphenol S)) OR (BPS)) OR (Bisphenol F)) OR (BPF)) OR (Bisphenol AF)) OR (BPAF)) OR (Bisphenol AP)) OR (BPAP)) OR (BPB)) AND ((((((((((((((((Thyroid) OR (Thyroid Problem)) OR (Thyroid Disease)) OR (Thyroid Disorder)) OR (Thyroid Dysfunction)) OR (Thyroid Hormone)) OR (T3)) OR (Diiodotyrosine)) OR (Calcitonin)) OR (T4)) OR (triiodothyronine)) OR (thyroxine)) OR (FT3)) OR (FT4)) OR (Free triiodothyronine)) OR (free thyroxine)).

Search strategies for all selected databases have been given in the Supplementary Materials (see Table S1). PRISMA (The Preferred Reporting Items for Systematic Reviews and meta-analysis) was used as a guide in this study (see Table S2). Two researchers independently conducted literature screening by following the PECO framework: Population: Adults and pregnant women exposed to bisphenols in Asia, Europe, and US; Exposure: bisphenol A and its alternatives (BPA, BPS, BPB, and BPF); Comparator: No exposure to bisphenols; and Outcome: HPT axis hormones (TSH, FT3, FT4, T3, T4, TT3, and TT4) of adults and pregnant women. The inclusion criteria were the following: (a) observational studies related to the objective; (b) studies with urinary bisphenols (A, S, F, B, and TBBPA) used as exposure; (c) human studies based on urinary Bisphenol exposure and blood HPT axis hormones; (d) studies providing beta, standard error (SE), and 95% confidence interval (CI) data; and (e) studies with literature quality scores higher than 7 (see Table S3).

The included articles provided this information: origin of the study, first author, year of publication, exposure duration, study design employed, age of the participants or the age of pregnancy, sample size, level of hormones, observed outcomes, population characteristics, demographics, bisphenol exposure assessment, HPT axis hormones detection in the general population, adjusted confounding variables, regression coefficient, and 95% confidence intervals. This refers to the fact that our study considered the pregnant population and adults and further divided the adults into males and females, as well as a group analysis by region.

### 2.2. Quality Assurance

We utilized the guidelines STROBE provided: the Newcastle Ottawa Scale (NOS) [3]. In addition, the 11-item standard suggested by AHRQ, the Agency for Healthcare Research and Quality, was used to evaluate the cross-sectional studies, as mentioned by [26].

### 2.3. Statistical Analysis

Bisphenol association with HPT axis hormones was evaluated by pooling the standard errors, regression coefficient (β), and 95% confidence interval (CI). We standardized the outcome units by using the formulas (Table S4) [27]. If the beta value is more than zero, it means the exposure will increase, and vice versa. The beta value means the increase in the outcome when the exposure increases by 1 unit, so the units of beta are the same as each outcome. Heterogeneity analysis used the Cochrane Q-test and *I*^2^ statistic, with an implication set at *p*-value < 0.1 or *I*^2^ ≥ 50%, indicating substantial statistical heterogeneity [28]. The DerSimonian–Laird random-effects model was employed to analyze the pooled results if heterogeneity was present, and this model considers both the differences within individual studies and the heterogeneity between different studies [29]. When the heterogeneity was insignificant, the PetoMante1–Haenszel fixed-effects model was utilized as an alternative.

Sensitivity analysis, performed through leave-one-out cross-validation [30], assessed the robustness. Bias in the literature was evaluated using the Begg and Egger tests [31,32], considering a *p*-value < 0.05 as statistically significant. Subgroup analysis addressed potential variability among studies. Forest plots are used to show all analyses [33] This meta-analysis used the “metagen” function from the “meta” package in R version 4.3.2 [34].

## 3. Results

### 3.1. Characteristics Overview

**Figure 1:**
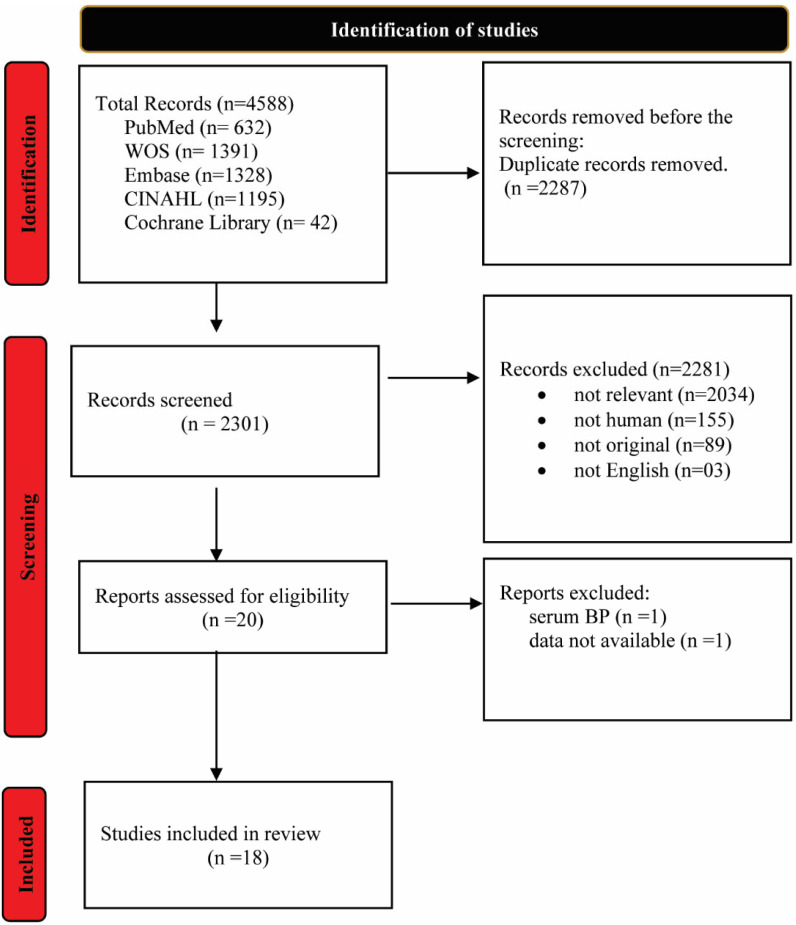
Literature screening flowchart.

A total of 4588 articles were obtained from the literature using five databases: Embase, PubMed, CINAHL, Web of Science, and Cochrane Library until January 2025. Eighteen studies from regions of Asia, the United States, and Europe were selected (Figure 1).

Eighteen eligible studies attempt to represent the study area’s population, with eight studies focusing on bisphenol exposure among pregnant women [12,13,35,36,37,38,39,40] and 10 studies focusing on bisphenol exposure in adults [23,24,25,41,42,43,44,45,46,47]. Ten studies were prospective cohorts, and the remaining eight were cross-sectional studies.

### 3.2. Quality of Included Studies

NOS scores demonstrate that the studies analyzed exhibit a high degree of quality. However, it should be noted that four of these studies were deemed to have insufficient follow-up, as indicated in Table S5. Furthermore, AHRQ conducted a quality assessment of the cross-sectional studies [26]. The assessment revealed that the majority of the five studies were deemed to be of good quality, as evidenced by the Supplementary Materials (Table S5). The NOS and AHRQ scores of the included studies ranged from 7 to 9. All these studies were rated as high quality according to the above criteria.

The eighteen included studies aimed to accurately represent the study area’s population, while 08 studies specifically assessed bisphenol (BP) exposure among pregnant women. Table 1 summarizes the studies on the association between bisphenol exposure and HPT axis hormones.

#### Systematic Review

A total of 18 studies were included in this systematic review, encompassing research conducted between 2011 and 2024 [12,13,23,24,25,35,36,37,38,39,40,41,42,43,44,45,46,47].

Aker et al. found a positive correlation between pregnant women’s TSH and BPA levels. In contrast to the stronger correlations shown at 24–28 weeks for BPA, 16–20 weeks may be more vulnerable to BPS and BPF exposure [37]. Previous research [13,38] has linked BPF exposure to increased FT3 and reduced TT4 in early pregnancy. BPA was associated with maternal blood TT4 in the first trimester, whereas there was no association between them in the third trimester [13]. Distinguished parental BPA exposure during the third trimester was connected to higher cord blood TSH in females and lower FT4 in male infants [39]. BPS, which replaced BPA, lowered FT4 to fewer than 15 weeks GA and increased TSH, especially at less than 15 weeks GA [36]. BPA levels in urine were positively connected with free T4 and negatively correlated with pregnant women’s TSH [12]. A prospective Chinese birth cohort study demonstrated that BPB, BPF, BPS, and TBBPA are toxic BPA alternatives, and those BPs are also harmful to human HPT axis hormones. Bisphenol’s effects on THs were sex-specific and nonlinear [22]. Another study concluded that exposure to bisphenols may affect the HPT axis hormones during pregnancy [40]. A cross-sectional study [42] found negative relationships between BPA and T3 in lower body mass index (BMI < 25.0 kg/m^2^) and T3 and T4 in higher BMI (>25.0 kg/m^2^). Urine BPA and serum TSH levels were positively correlated in lean people but not in obese people [41]. Both fT4 and TT4 were strongly correlated with BPF in men. Yue et al. also found connections between fT3 and BPF in women and TT4 and BPF [46]. In a Korean population study, BPA was connected to a 4.6% decrease in TSH, a negative association, and a similar relationship in both sexes [23]. BPA had a high inverse connection with TSH and a suggestive inverse link with total T4 in our adult sample [25]. In a study, urinary BPA was negatively connected with blood TSH in adults, males, and females, and directly correlated with serum-free triiodothyronine [43]. A study from China reported widespread exposure to bisphenols and HPT axis hormones in the general population, depending on age and gender [45]. Another study [47] revealed that BPA exposure is negatively associated with adult thyroxine. In another study, the researcher found a sex-specific association between exposure to EDCs and HPT axis hormones. It was identified that T3 and FT3 are positively associated with EDC in boys, and FT4 and T4 are negatively associated with bisphenols in girls [44]. These observations suggest that BPA may disrupt thyroid activity. Ten of these 18 studies were prospective cohorts lasting 1–5 years, while eight were cross-sectional. Beta and Standard error (SE) were included in all trials despite covariate adjustments. Bisphenol exposure and THs studies are controversial, but researchers constantly improve their methodology and findings.

## 4. Meta-Analysis Results

### 4.1. BP Exposure in Pregnant Women and HPT Axis Hormones

**Figure 2:**
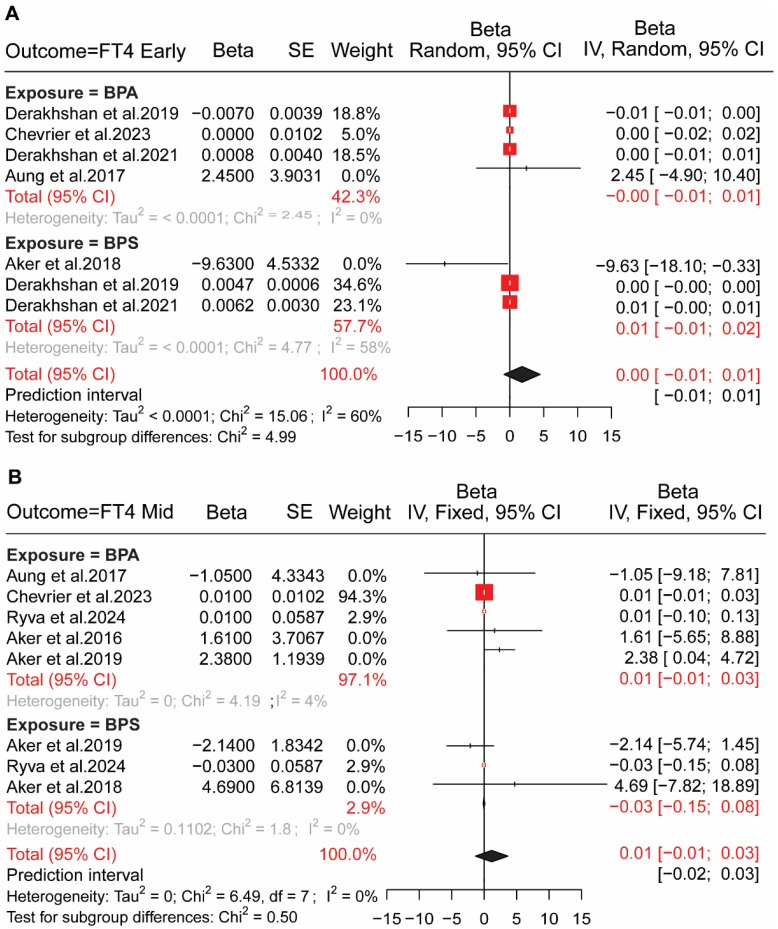
Association between bisphenol exposure and HPT axis hormones in early and mid-pregnancy. (**A**) Forest plot showing the association of bisphenol A (BPA) and bisphenol S (BPS) exposure with free thyroxine (FT4) levels during early pregnancy. Each red square represents the beta estimate for an individual study [12,13,36,38,39], with the size proportional to study weight. Horizontal lines indicate 95% confidence intervals (CI), and the black diamond represents the overall pooled effect. (**B**) Forest plot showing the association of BPA and BPS exposure with FT4 levels during mid-pregnancy. Results are presented for individual studies [12,13,35,36,37,40]. Red squares indicate study-specific beta estimates, horizontal lines indicate 95% CI, and black diamonds represent the pooled effect sizes. Red squares, individual study estimates; black diamonds, pooled effect estimates; horizontal lines, 95% CI; heterogeneity measures (Tau^2^, Chi^2^, I^2^) are shown below each panel.

**Figure 3:**
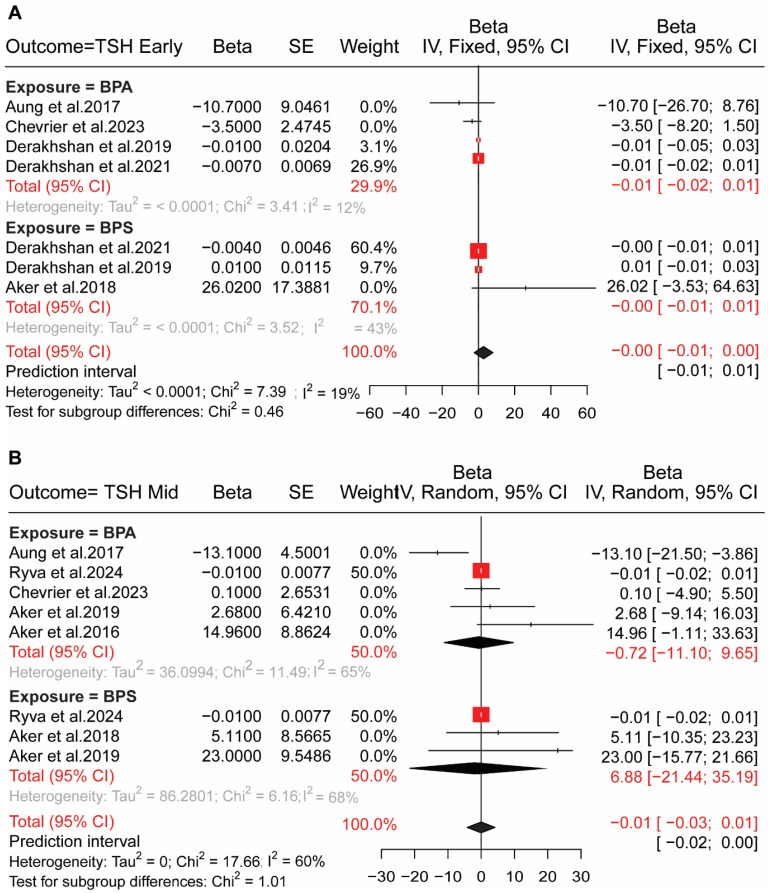
Association between exposure to bisphenol and HPT axis hormones in adults. (**A**) Forest plot showed the association of bisphenol A (BPA) and bisphenol S (BPS) exposure with thyroid-stimulating hormone (TSH) levels in early pregnancy. Each red square represents the beta estimate from an individual study [12,13,36,38,39], with the size proportional to the study weight. Horizontal lines indicate 95% confidence intervals (CI), and the black diamond represents the pooled effect estimate. (**B**) Forest plot showed the association of BPA and BPS exposure with TSH levels in mid-pregnancy. Results are presented for individual studies [12,13,35,36,37,40]. Red squares indicate study-specific beta estimates, horizontal lines represent 95% CI, and black diamonds denote the pooled effect sizes; heterogeneity measures (Tau^2^, Chi^2^, I^2^) are reported below each panel.

In early pregnancy, no association was observed between bisphenol exposures and HPT axis hormones (TSH, FT4, TT4) in early and mid-pregnancy. We determined the association between BPs (BPA, BPS) exposure and Free thyroxin (FT4) using a random effect model, where pooled results for seven included studies were (pooled β = −0.001; (95% CI: −0.011, 0.001)) in early pregnancy Figure 2A and eight studies included for mid-pregnancy in a fixed-effect model (pooled β = 0.010; (95% CI: −0.010, 0.030)) Figure 2B. We conducted a fixed-effect model for BPs exposure to the thyroid-stimulating hormone (TSH), and the results are (pooled β = −0.001; (95% CI: −0.010, 0.000)), (pooled β = −0.010; (95% CI: −0.030, 0.010)) for early and mid-pregnancy, respectively (Figure 3). We also determined the exposure to BPs and total thyroxine (TT4); a fixed-effect model was used for both early-pregnancy (β = 0.010; (95% CI: −0.030, −0.050)) with six studies and mid-pregnancy (β = −0.020; (95% CI: −0.110, 0.070)) with seven studies (Figure S1).

### 4.2. BPs Exposure in Adults and HPT Axis Hormones

**Figure 4:**
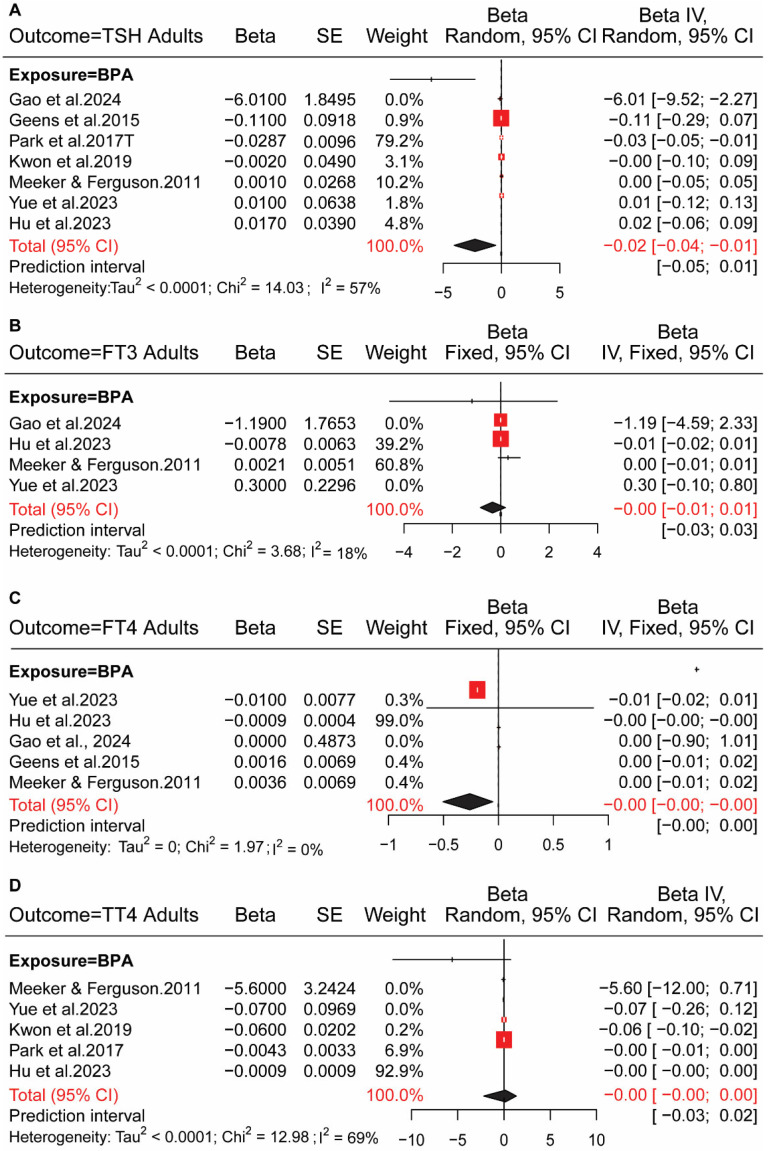
Association between exposure to bisphenols and HPT axis hormones in adults. (**A**) Forest plot showed the association between BPA exposure and thyroid-stimulating hormone (TSH) levels. Studies included [23,25,41,42,45,46,47]. (**B**) Forest plot showed the association between BPA exposure and free triiodothyronine (FT3) levels. Studies included [25,45,46,47]. (**C**) Forest plot showed the association between BPA exposure and free thyroxine (FT4) levels. Studies included [25,41,45,46,47]. (**D**) Forest plot showed the association between BPA exposure and total thyroxine (TT4) levels. Studies included [23,25,42,45,46]. Red squares, individual study beta estimates (with square size proportional to study weight); horizontal lines, 95% confidence intervals (CI); black diamonds, pooled effect estimates. Heterogeneity measures (Tau^2^, Chi^2^, I^2^) are reported below each subfigure.

A negative association was identified between BPA exposure and TSH in adults with estimated (β = −0.020; (95% CI: −0.040, −0.010)) and *I*^2^ = 57% (Figure 4A). We conducted a fixed-effect model due to low heterogeneity, *I*^2^ = 18% analyses. BPA did not have any correlation with FT3 (β = −0.001; 95% CI: −0.010, 0.010) (Figure 4B). The effect of BPA exposure on FT4 (β = −0.001; (95% CI: −0.001, −0.001)) was negatively associated with adults (Figure 4C). BPA with TT4 showed *I*^2^ = 69%, (β = −0.001; (95% CI: −0.001, 0.001)) (Figure 4D), BPA with TT3 showed *I*^2^ = 56%, (β = −0.001; (95% CI: −0.010, 0.001)) (Figure S2), which indicates no association between BPA exposure on both hormone levels in adults. Studies were not enough studies to conduct pooled analyses for combined bisphenol exposure.

**Figure 5:**
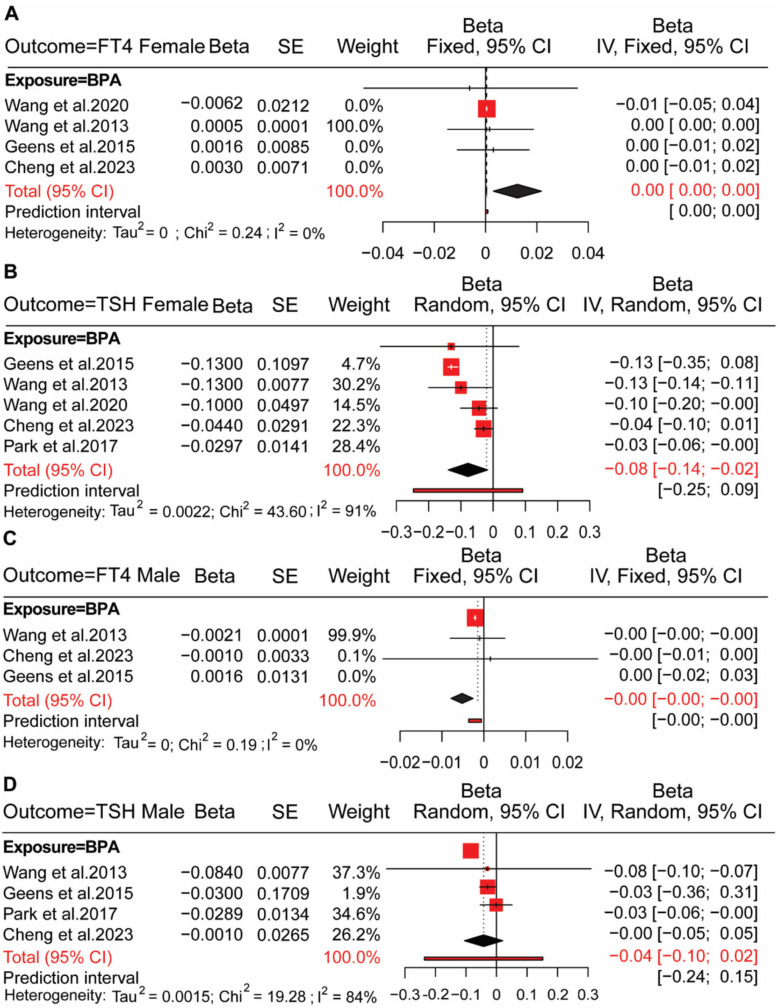
Association between exposure to bisphenols and HPT axis hormones in female and male subgroups. (**A**) Forest plot showed the association between BPA exposure and free thyroxine (FT4) levels in females. Studies included [24,41,43,44]. (**B**) Forest plot showed the association between BPA exposure and thyroid-stimulating hormone (TSH) levels in females. Studies included [23,24,41,43,44]. (**C**) Forest plot showed the association between BPA exposure and FT4 levels in males. Studies included [24,41,44]. (**D**) Forest plot showed the association between BPA exposure and TSH levels in males. Studies included [23,24,41,44]. Red squares, individual study beta estimates (with square size proportional to study weight); horizontal lines, 95% confidence intervals (CI); black diamonds, pooled effect estimates. Heterogeneity measures (Tau^2^, Chi^2^, I^2^) are reported below each subfigure.

The effect of bisphenol exposure on female HPT axis hormones matches four studies in the analysis of BPA and FT4. The heterogeneity of the test was 00.00%, and a fixed-effect model was used, where the overall effect size was β = 0.001 (95% CI: 0.001, 0.001), which shows that results are positively significant for BPA exposure in females’ FT4 (Figure 5A). We also conducted a random effect model with *I*^2^ = 91% to assess the association between females’ exposure to BPA and TSH, where the results were negative and significant. The overall effect size of studies was a −0.080 regression coefficient and 95% CI (−0.140, −0.020) (Figure 5B). BPA with male FT3 was estimated using a fixed-effect model (β = −0.001; 95% CI: −0.001, 0.001) and revealed a negative association (Figure 5C). Bisphenol exposure to male TSH matches studies with 84% heterogeneity. We conducted a random-effect model to test the association between exposure to BPA and male TSH. We concluded, with an estimated effect size (β = −0.040; 95% CI: −0.100, 0.020), that BPA has no significant relationship with male TSH (Figure 5D). There were minimal studies for other analogues of bisphenol exposure to HPT axis hormones; data were insufficient for a meta-analysis.

### 4.3. BPs Exposure and HPT Axis Hormones in Populations from Different Regions

Our meta-analysis included fourteen studies from eight countries. Based on geographical boundaries, we divided them into three main regions: Asia, America, and Europe. We could not analyze the European data as insufficient studies were available for meta-analysis. Whether the results are from the same population or different regions, it is difficult to see the significance of our regional comparison with the present results.

Based on evidence from Asia, we conducted a random-effect model for the association of BPA exposure to female TSH, while heterogeneity was 95%. The estimated effect size (β = −0.090; (95% CI: −0.220, 0.050)) denotes that there are no statistically significant results (Figure S3A). Adults exposed to BPA showed a negative association with TSH with a regression coefficient of −0.020 unit and 95% CI (−0.040, −0.010) using a fixed-effect model, where *I*^2^ = 0% (Figure S3B). For Asian adults, BPA exposure was not associated with TT3 (β = −0.001; 95% CI: −0.010, 0.000; *I*^2^ = 60%), denoting no statistically significant results (Figure S3C). Likewise, the random-effects model showed that BPA exposure was not associated with TT4 (β = −0.001; 95% CI: −0.001, 0.000), also indicating no statistically significant results (Figure S3D).

Based on evidence from the United States, according to pregnant women’s second-trimester data, eight studies were included in the fixed-effect model for BP exposure to FT4 with 0% heterogeneity. The resulting overall effect size (β = 0.010; 95%CI: −0.010, 0.030) showed no association (Figure S4A). We also conducted a random effect model for assessing the association of BPs with TSH in mid-pregnancy, which showed no significant association with the overall effect size (β = −0.010; 95% CI: −0.030.620, 0.010) with 60% heterogeneity (Figure S4B). Bisphenol exposure to TT4 for US pregnant women in mid-pregnancy (β = −0.010; (95% CI: −0.030.620, 0.010)) showed no association with 13% heterogeneity (Figure S5).

### 4.4. Small Study Effect and Sensitivity Analysis

The findings of Egger’s and Begg’s tests suggested no significant results for publication bias. Two analyses had publication bias, and there was an association with BPA exposure to adults TT3 and TT4, with different studies having different covariables (Figures S6–S10). We also performed a small study effect test using the trim and fill method (Figures S11–S16). Furthermore, the sensitivity analysis results demonstrated that omissions of the studies did not change the correlation of bisphenol exposure to HPT axis hormones. Each study made a balanced contribution to the analysis, and our findings are robust against specific risks, supporting the validity of our review (Figures S17–S22).

## 5. Discussion

This study aimed to determine the association between bisphenol exposure and serum HPT axis hormone levels in adults and pregnant women. Eighteen studies were eligible to be included. Our study reveals the diverse bisphenol (BP) exposure levels in various populations and regions. We compared these levels with values reported in other pieces of the literature and analyzed the reasons for differences in exposure among different population groups. Our findings suggest that combined bisphenol exposure could affect HPT axis hormones in adult males, females, and pregnant women.

Thyroid hormones are key to growth, development, and metabolism, especially a child’s neurodevelopment during pregnancy. They regulate metabolism and play a role in the normal growth of adults. Widespread human exposure to bisphenols occurs through the use of plastic materials and food packaging products. In recent practice, industries have replaced BPA with alternatives such as BPS, BPB, BPAP, and BPF. We are focusing on bisphenol A, and its alternatives still have a high risk of HPT axis hormone abnormality.

Our study indicates that no significant relationship exists between bisphenol metabolites and HPT axis hormones during early and mid-pregnancy. Previously, it was also reported that the combined exposure to bisphenols (BPs) does not significantly impact THs during early and mid-pregnancy, but individual metabolites of bisphenol A (BPA) have different roles on the HPT axis in pregnant women [37,38].

Our findings indicate that BPA is negatively associated with TSH and FT4, and there were no significant results for FT3, TT3, and TT4 in adults; our gender-based analysis demonstrated that there is a negative relationship between BPA exposure with TSH where a positive correlation towards FT4 in female, on another side there were no significant results found for BPA exposure to TSH in adult males, and BPA and FT4 were negatively associated in adult males. There were a small number of studies on this association in subgroup analysis, so our findings are conflicting. The study [23] found a negative relationship between urinary BPA concentrations and adults’ TSH levels in the Korean NEHS from 2012–2014. Another study [24] reported a negative association between urinary concentrations of BPA, elevated levels of FT3, and reduced levels of TSH among Chinese adults. In the previous investigation, urinary BPA was negatively associated with serum TT3 concentrations in adult females. According to [45], a negative association was observed between BPA and FT4 and TSH levels in girls. Similarly, the Thai NHES 2009 study [48] reported a negative association between blood BPA concentrations and free T4 in men. Our results are not supported due to the limited data. It is suggested that there is no association, and covariant factors may affect the result. The findings of a prior meta-analysis comprising 11 studies showed a negative correlation between the concentration of BPA and FT4 and TSH in men. The impact of BPA on HPT axis hormone levels exhibited significant gender-based variations. Specifically, the concentration of BPA displayed a positive correlation with FT4 levels in females [49]. The observed gender difference may be associated with variations in the androgen-related metabolism of BPA [48]. Previous investigations have shown that many people can be exposed to BPA and its metabolites.

Furthermore, evidence links BPs exposure to alterations in HPT axis hormone levels [50]. TH restrains metabolic activities essential for suitable growth, development, and adult metabolism [51,52].

Thyroid hormones are synthesized within the thyroid gland, and the pituitary glands produce thyroid-stimulating hormone, which regulates the thyroid hormones. When TSH is present, it leads to the stimulation of thyrocytes. T4, also known as thyroxine, and T3, which stands for triiodothyronine, are two critical hormones regulating various physiological processes in the human body. BPA inhibits thyroid function primarily through its TR-antagonistic effect. Many BPA metabolites, for example, bisphenol F (BPF) and bisphenol S (BPS), are being used more due to public health concerns. Due to their structural similarity to BPA and T3, these bisphenols may affect thyroid function. Since bisphenols were recently introduced, little research has examined their effects on thyroid dysfunction. According to [53], the binding affinity between BPA, TBG, and TTR is relatively low.

In contrast, it has been observed that metabolites of BPA, such as tetrachlorinated bisphenol (TCBPA) or tetra-brominated bisphenol (TBBPA), have a heightened similarity [54]. Bisphenols can form a complex with thyroid hormone receptors (TRs), specifically the beta isoform of TR (TRβ), and show antagonistic properties [10,55]. There is a potential for BPA and its metabolites, BPF and BPS, to have a direct impact on the thyroid gland, as indicated by the recent finding that in children, there was a negative correlation between urine BPA levels and thyroid hormone [56]. BPA could form a complex with thyroid hormone receptors (TR), namely the β isoform of TR (TRβ), and exhibits antagonistic properties [10,55]. There is a potential for BPA and its metabolites, BPF and BPS, to have a direct impact on the thyroid gland, as observed in a recent study on children; there was a negative correlation between urine BPA levels and thyroid hormone [56]. The study [11] also indicates that bisphenol A (BPA) can potentially interfere with thyroid functions.

Our research has the following strong points. We are the first to observe that bisphenol and its analogs (BPA, BPS, BPF, BPB, and TBBPA) influence adults, men, women, and pregnant women (first and second trimester) HPT axis hormones levels and also examined at the regional level. Secondly, the Trim and Fill method was used to identify the publication bias for the outcome, ensuring the results’ correctness. However, some restrictions still occurred in this investigation. Firstly, post-birth exposure cannot be neglected; pregnancy is a vital time for neurodevelopmental problems; we did not verify the values for the third trimester. We have incorporated that our meta-analysis did not include other forms of phenols or endocrine-disrupting chemicals. Although the studies accounted for different factors, we included beta and standard errors in all the research. In enough studies, we have not observed serum samples for bisphenol exposure. Although contradictory results exist, experts always work to improve their research techniques and findings on the relationship between bisphenol exposure and THs.

This meta-analysis may be used as preliminary research to identify potential relationships, and its results should be carefully read. More research utilizing novel methodologies is required to investigate the relationship between BPs exposure and THs.

## 6. Conclusions

Our data showed a substantial positive relationship between BPs and FT4 in males, and BPA was negatively associated with TSH in females. Furthermore, our findings show that exposure to BPA reduces FT4 and TSH levels in adults. Furthermore, no association was found between bisphenol exposure and TSH, FT4, and TT4 levels in pregnant women during early and mid-pregnancy. The impact of gender variations on bisphenol exposure to THs levels is significant, and a large-scale study is required to support these conclusions. A large-scale cohort study or cross-sectional study design can provide more robust evidence focusing on the impact of bisphenol exposure on HPT axis hormones in pregnant women and adults.

## Figures and Tables

**Table 1:** Summary of the articles on the association between exposure to bisphenol and HPT axis hormones.

Author Name	Year	Research Design	Sample Size	Country	Stage/Gender	Bisphenol Type (BP)	Sample	Detection Method	Outcome	Adjusted Variables
Aker et al. [37]	2019	Cohort	602	United States	2nd trimester	A, S, F	Urine	HPLC-ID-MS/MS	TSH, FT4, T4	Maternal age (MA), study visit (SV), Specific gravity (SG), BMI at 1st visit, the passive smoking exposure (PS) in hours, and a socio-economic variable (SEV)
Derakhshan et al. [38]	2019	Cohort	1996	Sweden	1st trimester	A, S, F	Urine	LC-MS/MS	TSH, FT4, FT3, TT4, TT3	MA, thyroid peroxidase antibodies (TPa), thyroglobulin antibodies (Tba), gestational age (GA), human chorionic gonadotropin, urinary creatinine (UC), smoking, BMI and SEV
Derakhshan et al. [39]	2021	Cohort	1267	The Netherlands	1st trimester	A, S	Urine	HPLC-ESI-MS/MS	TSH, FT4, TT4	GA, MA, TPa, human chorionic gonadotropin, UC, BMI, education level, ethnicity, smoking status, and parity
Aker et al. [36]	2018	Cohort	439	Boston, MA	1st, 2nd, 3rd trimesters	S	Urine	ID-LC-MS/MS	TSH, FT4, T4, T3	SG, SV, BMI, MA and GA, and insurance provider.
Kwon et al. [42]	2020	Cross-Sectional	5108	Korea	Adults	A	Urine	UPLC-MS/MS	TSH, T4, T3	Smoking status, alcohol consumption, exercise, SV, MEHHP, MEOHP, MECPP, MnBP, MBzP, age and SEV.
Chevrier et al. [13]	2013	Cohort	335	United States	1st, 2nd trimester	A	Urine	HPLC- ID-MS/MS	TSH, FT4, TT4	MA, iodine intake, and hexachlorobenzene and polychlorinated biphenyl, SEV, drugs in pregnancy
Geens et al. [41]	2015	Cohort	151	Belgium	Male, female, and Total adults	A	Urine	GC–MS system	TSH, FT4	Age and weight loss
Yue et al. [46]	2023	Cross-Sectional	177	China	Adults	A, S, F	Urine	UPLC-MS/MS	TSH, FT4, FT3, TT4, TT3	Age, sex, BMI, smoking, urinary iodine, and SEV.
Wang et al. [43]	2020	Cohort	555	China	Female	A	Urine	HPLC-MS/MS	TSH, FT4	UC, MA, PS, GDM (yes/no), GA and SEV
Aker et al. [35]	2016	Cohort	106	United States	2nd trimester	A	Urine	HPLC-ID-MS/MS	TSH, FT4, FT3	SG, SV, MA, BMI, and SEV
Aung et al. [12]	2017	Cohort	439	Boston, MA	1st, 2nd, 3rd trimester	A	Urine	ID-LC-MS/MS	TSH, FT4, FT3, TT4, TT3	SG, GA, MA, BMI and SEV
Park et al. [23]	2017	Cross-Sectional	5870	Korea	Male, female, and total adults	A	Urine	LLE-UPLC-MS/MS	TSH, TT4, TT3	Age, BMI, sex, PS, UC, and SEV
Meeker & Ferguson [25]	2011	Cross-Sectional	1675	United States	Adults	A	Urine	HPLC-ID-MS/MS	TSH, FT4, FT3, TT4, TT3	Age, BMI, sex, ethnicity, BMI, UC, and iodine
Wang et al. [24]	2013	Cross-Sectional	3394	China	Male and female	A	Urine	HPLC-MS/MS	TSH, FT4, FT3	UC, age, BMI, SEV, alcohol, triglycerides, HDLC, LDL-C, Tba, and TPa
Cheng et al. [44]	2023	Cross-Sectional	2911	United States	Male and female	A	Urine	SPE-HPLC-MS/MS	TSH, FT4, FT3, TT4, TT3	Age, sex, race, education, poverty level, BMI, serum cotinine, UI, hypertension, DM, and UC
Gao et al. [47]	2024	Cross-Sectional	2385	United States	Adults	A	Urine	SPE-HPLC-MS/MS	TSH, FT4, FT3, T4, T3	Age, BMI, gender, smoking, drinking, race, education, medication history, marital status, PIR, and UC level
Hu et al. [45]	2023	Cross-Sectional	150	China	Adults	A	Urine	UPLC-MS/MS	TSH, FT4, FT3, TT4, TT3	Age, BMI, gender, education levels, smoking, alcohol, UC, occupation
Ryva et al. [40]	2024	Cohort	302	Chicago	2nd trimester	A & S	Urine	ID-MS/MS	TSH, FT4, TT4	Age, diet, pre-pregnancy BMI, stress, smoking status, parity, race, GA

Note: 1st trimester—gestational week 1–16 weeks, 2nd trimester—gestational week 17–28, 3rd trimester—>28 gestational weeks, adults age >12 years, free thyroxine, FT3, TSH—thyroid stimulating hormone, FT4, free triiodothyronine, TT3—total triiodothyronine, TT4—total thyroxine, SG—specific gravity, SEV—socio-economic variable, GA—gestational age, BMI—body mass index, UC—urinary creatinine, HPLC-MS/MS—high-performance liquid chromatography, ID-MS/MS: isotope dilution tandem mass spectrometry, LC-MS/MS—liquid chromatography-tandem mass spectrometry, LLE—liquid-liquid extraction, HPLC-ESI-MS/MS—high performance liquid chromatography-electrospray ionization-tandem mass spectrometry, UPLC-MS—ultra-performance liquid chromatography-mass spectrometry, ID-LC-MS/MS—isotope dilution liquid chromatography-tandemA mass spectrometry, UPLC-MS—ultra-performance liquid chromatography-mass spectrometry, GC–MS system, gas chromatography-mass spectrometry, SPE-HPLC-MS/MS: solid phase extraction combined with high-performance liquid chromatography, MEHHP: mono(2-ethyl-5-hydroxyphenyl) phthalate, MEOHP-mono(2-ethyl-5-oxohexyl) phthalate, MECPP-mono(2-ethyl-5-carboxymethyl) phthalate, MnBP—mono n-butyl phthalate; MBzP-mono benzyl phthalate. Other details of units, LOD, concentrations, and GM are given in Table S6.

## Data Availability

All data, codes, or any information can be obtained through the corresponding authors.

## Supplementary Materials

The following supporting information can be downloaded at https://www.mdpi.com/article/10.3390/toxics13100836/s1, Table S1: Search Strategy for all selected databases; Table S2: PRISMA Checklist 2020; Table S3: PECO Statement; Table S4: Standardized units and corresponding conversion formulas for thyroid function analytes; Table S5: Detailed Newcastle-Ottawa Scale of each included cohort study; Table S6: study characteristics (concentrations); Figure S1: Association between bisphenols exposure to total thyroxine (TT4) in pregnant women; Figure S2: Association between bisphenols exposure to total triiodothyronine in adults and trim fill results; Figure S3: Association between bisphenols exposure to HPT axis hormones in Asian adults; Figure S4: Association between bisphenols exposure to HPT axis hormones in US pregnant women; Figure S5: Association between bisphenols exposure to total thyroxine in mid pregnancy; Figure S6: Publication bias for pregnant women; Figure S7: Publication Bias for adult studies; Figure S8: Publication Bias for female and male gender; Figure S9: publication Bias for Asia region; Figure S10: Publication bias for US region; Figure S11: trim fill analysis results in early pregnancy; Figure S12: trim fill analysis results in mid pregnancy; Figure S13: trim fill analysis results in adults; Figure S14: trim fill analysis results in both gender; Figure S15: trim fill analysis results for Asia region; Figure S16: trim fill analysis results for US region; Figure S17: leave one out method results for pregnant women in early pregnancy; Figure S18: leave one out method results for pregnant women in early pregnancy; Figure S19: leave one out method results for adults; Figure S20: leave one out method results for female and male subgroups; Figure S21: leave one out method results for Asia region; Figure S22: leave one out method results for US region.

## Abbreviations

TSH—Thyroid Stimulating Hormone, T_3_—Triiodothyronine, FT_3_—Free Triiodothyronine, T_4_—Thyroxine, FT_4_—Free Thyroxine, TT_3_—Total triiodothyronine, TT_4_—Total Thyroxine, HPT axis hormone—Hypothalamic–Pituitary–Thyroid axis hormones, THs—thyroid hormones, Korean National Examination Health Survey (KNEHS). BPA—Bisphenol A, BPB—Bisphenol B, BPAP—Bisphenol A phosphate, BPAF—Bisphenol AF, BPS—bisphenol S, TBBPA—Tetrabromobisphenol A, BPF—Bisphenol F.
