# Supplementary material for: Association of Bisphenol Exposure and Serum Hypothalamic–Pituitary–Thyroid Axis Hormone Levels in Adults and Pregnant Women: A Systematic Review and Meta-Analysis
Source: Toxics. 2025 Sep 30;13(10):836. doi: 10.3390/toxics13100836 (PMC12568303; doi:10.3390/toxics13100836)
Supplement: Supplementary file 1 [file toxics-13-00836-s001.zip › toxics-3869578-supplementary.pdf]

Systematic Review

Association of Bisphenol Exposure and Serum Hypothalamic–Pituitary–Thyroid Axis Hormone Levels in Adults and Pregnant Women: A Systematic Review and Meta-Analysis

Mazhar Sultan [1,2,†], Xuan Ma [3,†], Qiurun Yu [1,2], Francis Manyori Bigambo [4], Yufeng Tang [5], Natasha Chitakwa [1,2], Farah Kafauit [6], Qinrou Chen [1,2], Quanquan Guan [1,2,*] and Yankai Xia [1,2,*]

1 State Key Laboratory of Reproductive Medicine and Offspring Health, Center for Global Health, School of Public Health, Nanjing Medical University, Nanjing 211166, China; mazharsa03@njmu.edu.cn (M.S.)
2 Key Laboratory of Modern Toxicology of Ministry of Education, School of Public Health, Nanjing Medical University, Nanjing 211166, China
3 The Affiliated Wuxi Center for Disease Control and Prevention of Nanjing Medical University, Wuxi Center for Disease Control and Prevention, Wuxi Medical Center, Nanjing Medical University, Nanjing 210000, China; maxuan@njmu.edu.cn
4 Children's Hospital of Nanjing Medical University, Nanjing 211166, China
5 Department of Policy and Public Management, Zanvyl Krieger School of Arts and Sciences, Johns Hopkins University, Washington, DC 20001, USA
6 Department of Clinical Pharmacology, Sir Run Run Hospital, Nanjing Medical University, Nanjing 211166, China
* Correspondence: yankaixia@njmu.edu.cn (Y.X.); guanqq@njmu.edu.cn (Q.G.)
† These authors contributed equally to this work and are co-first authors.

List of Tables and Figures

Table S1. Search Strategy for all selected databases

Table S2. PRISMA Checklist 2020

Table S3. PECO Statement

Table S3. PECO Statement.

P — Population

- Adults and pregnant women exposed to bisphenols in Asia , Europe, and US

E — Exposure

- bsphenol A and its alternatives (BPA, BPS, BPB, and BPF)

C — Comparator

- No exposure to bisphenols

O — Outcome

- Thyroid Hormones (TSH, FT3, FT4, T3, T4, TT3, and TT4) of adults and pregnant women

Table S4. Standardized units and corresponding conversion formulas for thyroid function analytes

Table S3. PECO Statement.

P — Population

- Adults and pregnant women exposed to bisphenols in Asia , Europe, and US

E — Exposure

- bsphenol A and its alternatives (BPA, BPS, BPB, and BPF)

C — Comparator

- No exposure to bisphenols

O — Outcome

- Thyroid Hormones (TSH, FT3, FT4, T3, T4, TT3, and TT4) of adults and pregnant women

Table S4. Standardized units and corresponding conversion formulas for thyroid function analytes.

Analyte	Reported Unit(s)	Conversion Formula	Standard Unit
**TSH**	mIU/L	No Conversion	µIU/mL
**FT3**	pmol/L	1 pmol/L × 0.651	pg/mL
**FT3**	ng/dL	1 ng/dL ×10	pg/mL
**FT4**	pmol/L	1 pmol/L × 0.0777	ng/dL
**TT3**	nmol/L	1 nmol/L × 65.1	ng/dL
**TT3**	µg/dL	1 nmol/L × 1000	ng/dL
**TT4**	nmol/L	1 nmol/L × 0.0777	µg/dL
**TT4**	µg/mL	1 µg/mL ×10	µg/dL

Table S5. Detailed Newcastle-Ottawa Scale of each included cohort study

Table S2. PRISMA Checklist 2020.

TITLE

Title	1	Identify the report as a systematic review.	1
		**ABSTRACT**	
Abstract	2	See the PRISMA 2020 for Abstracts checklist.	2
		**INTRODUCTION**	
Rationale	3	Describe the rationale for the review in the context of existing knowledge.	3,4
Objectives	4	Provide an explicit statement of the objective(s) or question(s) the review addresses.	4
		**METHODS**	
Eligibility criteria	5	Specify the inclusion and exclusion criteria for the review and how studies were grouped for the syntheses.	5
Information sources	6	Specify all databases, registers, websites, organisations, reference lists and other sources searched or consulted to identify studies. Specify the date when each source was last searched or consulted.	4
Search strategy	7	Present the full search strategies for all databases, registers and websites, including any filters and limits used.	S1
Selection process	8	Specify the methods used to decide whether a study met the inclusion criteria of the review, including how many reviewers screened each record and each report retrieved, whether they worked independently, and if applicable, details of automation tools used in the process.	4
Data collection process	9	Specify the methods used to collect data from reports, including how many reviewers collected data from each report, whether they worked independently, any processes for obtaining or confirming data from study investigators, and if applicable, details of automation tools used in the process.	5
Data items	10a	List and define all outcomes for which data were sought. Specify whether all results that were compatible with each outcome domain in each study were sought (e.g. for all measures, time points, analyses), and if not, the methods used to decide which results to collect.	4,7,8
	10b	List and define all other variables for which data were sought (e.g. participant and intervention characteristics, funding sources). Describe any assumptions made about any missing or unclear information.	4,,8,9
Study risk of bias assessment	11	Specify the methods used to assess risk of bias in the included studies, including details of the tool(s) used, how many reviewers assessed each study and whether they worked independently, and if applicable, details of automation tools used in the process.	4,6
Effect measures	12	Specify for each outcome the effect measure(s) (e.g. risk ratio, mean difference) used in the synthesis or presentation of results.	6
Synthesis methods	13a	Describe the processes used to decide which studies were eligible for each synthesis (e.g. tabulating the study intervention characteristics and comparing against the planned groups for each synthesis (item #5)).	4
	13b	Describe any methods required to prepare the data for presentation or synthesis, such as handling of missing summary statistics, or data conversions.	N/A
	13c	Describe any methods used to tabulate or visually display results of individual studies and syntheses.	5
	13d	Describe any methods used to synthesize results and provide a rationale for the choice(s). If meta-analysis was performed, describe the model(s), method(s) to identify the presence and extent of statistical heterogeneity, and software package(s) used.	5
	13e	Describe any methods used to explore possible causes of heterogeneity among study results (e.g. subgroup analysis, meta-regression).	5
	13f	Describe any sensitivity analyses conducted to assess robustness of the synthesized results.	5
Reporting bias assessment	14	Describe any methods used to assess risk of bias due to missing results in a synthesis (arising from reporting biases).	5
Certainty assessment	15	Describe any methods used to assess certainty (or confidence) in the body of evidence for an outcome.	5
		**RESULTS**	
Study selection	16a	Describe the results of the search and selection process, from the number of records identified in the search to the number of studies included in the review, ideally using a flow diagram.	5
	16b	Cite studies that might appear to meet the inclusion criteria, but which were excluded, and explain why they were excluded.	5
Study characteristics	17	Cite each included study and present its characteristics.	7,8 (Table1)
Risk of bias in studies	18	Present assessments of risk of bias for each included study.	11, S1
Results of individual studies	19	For all outcomes, present, for each study: (a) summary statistics for each group (where appropriate) and (b) an effect estimates and its precision (e.g. confidence/credible interval), ideally using structured tables or plots.	10-14

Results of syntheses	20a	For each synthesis, briefly summarise the characteristics and risk of bias among contributing studies.	10-14
	20b	Present results of all statistical syntheses conducted. If meta-analysis was done, present for each the summary estimate and its precision (e.g. confidence/credible interval) and measures of statistical heterogeneity. If comparing groups, describe the direction of the effect.	10-14
	20c	Present results of all investigations of possible causes of heterogeneity among study results.	10-14
	20d	Present results of all sensitivity analyses conducted to assess the robustness of the synthesized results.	Figure S11-S16
Reporting biases	21	Present assessments of risk of bias due to missing results (arising from reporting biases) for each synthesis assessed.	Figure S6-S10
Certainty of evidence	22	Present assessments of certainty (or confidence) in the body of evidence for each outcome assessed.	Figure S17-S22
		**DISCUSSION**	
Discussion	23a	Provide a general interpretation of the results in the context of other evidence.	17
	23b	Discuss any limitations of the evidence included in the review.	19
	23c	Discuss any limitations of the review processes used.	19
	23d	Discuss implications of the results for practice, policy, and future research.	19
		**OTHER INFORMATION**	
Registration and protocol	24a	Provide registration information for the review, including register name and registration number, or state that the review was not registered.	22
	24b	Indicate where the review protocol can be accessed, or state that a protocol was not prepared.	22
	24c	Describe and explain any amendments to information provided at registration or in the protocol.	22
Support	25	Describe sources of financial or non-financial support for the review, and the role of the funders or sponsors in the review.	17
Competing interests	26	Declare any competing interests of review authors.	17
Availability of data, code, and other materials	27	Report which of the following are publicly available and where they can be found: template data collection forms; data extracted from included studies; data used for all analyses; analytic code; any other materials used in the review.	17

From: For more information, visit: http://www.prisma-statement.org/, accessed on: 23 September 2025

Table S3. PECO Statement.

P **Population**

- Adults and pregnant women exposed to bisphenols in Asia , Europe, and US

E **Exposure**

- bsphenol A and its alternatives (BPA, BPS, BPB, and BPF)

C **Comparator**

- No exposure to bisphenols

O **Outcome**

- Thyroid Hormones (TSH, FT3, FT4, T3, T4, TT3, and TT4) of adults and pregnant women

Table S4. Standardized units and corresponding conversion formulas for thyroid function analytes.

Analyte	Reported Unit(s)	Conversion Formula	Standard Unit
**TSH**	mIU/L	No Conversion	µIU/mL
**FT3**	pmol/L	1 pmol/L × 0.651	pg/mL
**FT3**	ng/dL	1 ng/dL ×10	pg/mL
**FT4**	pmol/L	1 pmol/L × 0.0777	ng/dL
**TT3**	nmol/L	1 nmol/L × 65.1	ng/dL
**TT3**	µg/dL	1 nmol/L × 1000	ng/dL

**TT4**	nmol/L	1 nmol/L × 0.0777	µg/dL
**TT4**	µg/mL	1 µg/mL ×10	µg/dL

Table S5. Detailed Newcastle-Ottawa Scale of each included cohort study.

**Study (First Author)**	**Study Design**	**Selection**				**Comparability**	**Outcome/Exposure**			**Score**
		**Representa-tiveness of exposed**	**Selection of non-exposed**	**Ascertain-ment of exposure**	**Outcome, not initial present**	**Based on design and analysis**	**Assessment of outcome**	**Long follow-up for outcome**	**Adequate cohort follow-up**	
**Aker et al. 2019**	**Cohort**	1	1	1	1	2	1	0	1	08
**Derakhshan et al. 2019**	Cohort	1	1	1	1	2	1	1	0	08
**Derakhshan et al. 2020**	Cohort	1	1	1	1	2	1	1	0	08
**Aker et al. 2018**	Cohort	1	1	1	1	2	1	0	0	07
**Chevrier et al. 2023**	Cohort	1	1	1	1	2	1	0	1	08
**Geens et al. 2015**	Cohort	1	1	1	1	2	1	1	1	09
**Wang et al. 2020**	Cohort	1	1	1	1	2	1	1	0	08
**Aker et al. 2016**	Cohort	1	1	1	1	2	1	0	1	08
**Aung et al. 2017**	Cohort	1	1	1	1	2	1	0	1	08
**Ryva et al. 2024**	Cohort	1	1	1	1	1	1	0	1	08
**Study (First Author)**	**Study Design**	**Representativeness of the sample**	**sample size**	**Non-respondents**	**Ascertainment of Exposure**	**Based on design and analysis**	**Assessment of outcome**	**Statistical test**		
**Kwon et al. 2020**	Cross-Sectional	1	1	1	1	2	2	1		09
**Yue et al. 2023**	Cross-Sectional	0	0	1	1	2	2	1		07
**Park et al. 2017**	Cross-Sectional	1	1	1	1	2	1	1		09
**Meeker & Ferguson. 2011**	Cross-Sectional	1	1	1	1	2	1	1		09
**Wang et al. 2013**	Cross-Sectional	1	1	1	1	2	1	1		09
**Cheng et al. 2023**	Cross-Sectional	1	1	1	1	2	1	1		09
**Gao et al. 2024**	Cross-Sectional	1	1	1	1	2	2	1		09
**Hu et al. 2023**	Cross-Sectional	1	0	1	1	1	1	1		07

1) Was follow-up long enough for outcomes to occur: 1, duration of follow-up >=3 years; 0 if duration of follow-up < 3 years.

2) Loss to follow-up rate: 1, complete follow-up or loss to follow-up rate <=20 %; 0, follow-up rate < 80% or no description of those lost.

Very Good Studies: 9-10 points; Good Studies: 7-9 points; Satisfactory Studies; 5-6 points; Unsatisfactory Studies: 0 to 4 points

Table S6. study characteristics (concentrations)

No table of figures entries found.

Fig S 1: Association between bisphenols exposure to total thyroxine (TT4) in pregnant women 12
Fig S 2: Association between bisphenols exposure to total triiodothyronine in adults and trim fill results 13
Fig S 3: Association between bisphenols exposure to HPT axis hormones in Asian adults 14

Fig S 4: Association between bisphenols exposure to HPT axis hormones in US pregnant women 15
Fig S 5: Association between bisphenols exposure to total thyroxine in mid pregnancy 16
Fig S 6: Publication bias for pregnant women 17
Fig S 7: Publication Bias for adult studies 18
Fig S 8: Publication Bias for female and male gender 19
Fig S 9: publication Bias for Asia region 20
Fig S 10: Publication bias for US region 20
Fig S 11: trim fill analysis results in early pregnancy 21
Fig S 12: trim fill analysis results in mid pregnancy 22
Fig S 13: trim fill analysis results in adults 23
Fig S 14: trim fill analysis results in both gender 24
Fig S 15: trim fill analysis results for Asia region 25
Fig S 16: trim fill analysis results for US region 26
Fig S 17: leave one out method results for pregnant women in early pregnancy 27
Fig S 18: leave one out method results for pregnant women in early pregnancy 28
Fig S 19: leave one out method results for adults 29
Fig S 20: leave one out method results for female and male subgroups 30
Fig S 21: leave one out method results for Asia region 31
Fig S 22: leave one out method results for US region 32

Characteristics overview

The 18 eligible included studies attempt to represent the population of the study area, with 8 studies focusing on Bisphenol exposure among pregnant women and 10 studies were based on adults population [12,13,18,23,24,35-47]. Ten studies were prospective cohorts, and the remaining 08 were cross-sectional studies.

Inclusion Criteria

(a) Cohort, case-control, and cross-sectional epidemiological research. (b) The exposure factor was urinary Bisphenols (A, S, F, B, and TBBPA). c) The study found a link between urinary Bisphenol exposure and serum Hypothalamic-Pituitary-Thyroid axis hormones (HPT axis hormones; TSH, T3, T4, FT3, FT4, TT3, TT4). (d) The study provided Beta, SE, and 95% CI data. e) The literature quality score is higher than 7.

Exclusion Criteria

(a) studies that are not exposed to bisphenols (b) All types of reviews, protocols, and editorial meetings. c) clinical trials excluded age limits less than 12 years old e) all studies based on animal models.

Table S1. Search Strategy for all selected databases.

**PUBMED: 632 results retrieved from interception to January,08,2025**	
**#1**	((((((((((Bisphenol A) OR (BPA)) OR (Bisphenol S)) OR (BPS)) OR (Bisphenol F)) OR (BPF)) OR (Bisphenol AF)) OR (BPAF)) OR (Bisphenol AP)) OR (BPAP)) OR (BPB))
#2	(((((((((((((((Thyroid) OR (Thyroid Problem)) OR (Thyroid Disease)) OR (Thyroid Disorder)) OR (Thyroid Dysfunction)) OR (Thyroid Hormone)) OR (T3)) OR (Diiodotyrosine)) OR (Calcitonin)) OR (T4)) OR (triiodothyronine)) OR (thyroxine)) OR (FT3)) OR (FT4)) OR (Free triiodothyronine)) OR (free thyroxine))
#3	#1 AND #2
**CINAHL: 1328 results retrieved from interception to January,08,2025**	
S1 query	(Thyroid OR Thyroid Problem OR Thyroid Disease OR Thyroid Disorder OR Thyroid Dysfunction OR Thyroid Hormone OR T3 OR Diiodotyrosine OR Calcitonin OR T4 OR triiodothyronine OR thyroxine OR FT3 OR FT4 OR Free triiodothyronine OR free thyroxine) AND (Bisphenol A OR BPA OR Bisphenol S OR BPS OR Bisphenol F OR BPF OR Bisphenol AF OR BPAF OR Bisphenol AP OR BPAP OR BPB)
**Embase: 1195 results retrieved from interception to January,08,2025**	
#1	(Bisphenol A) OR (BPA) OR (Bisphenol S) OR (BPS) OR (Bisphenol F) OR (BPF) OR (Bisphenol AF) OR (BPAF) OR (Bisphenol AP) OR (BPAP) OR (BPB)
#2	(Thyroid) OR (Thyroid Problem) OR (Thyroid Disease) OR (Thyroid Disorder) OR (Thyroid Dysfunction) OR (Thyroid Hormone) OR (T3) OR (Diiodotyrosine) OR (Calcitonin) OR (T4) OR (triiodothyronine) OR (thyroxine) OR (FT3) OR (FT4) OR (Free triiodothyronine) OR (free thyroxine)
#3	#1 AND #2
**Web of Science: 1391 results retrieved from interception to January,08,2025**	
#1	TS= (Bisphenol A OR BPA OR Bisphenol S OR BPS OR Bisphenol F OR BPF OR Bisphenol AF OR BPAF OR Bisphenol AP OR BPAP OR BPB)
#2	TS= (Thyroid OR Thyroid Problem OR Thyroid Disease OR Thyroid Disorder OR Thyroid Dysfunction OR Thyroid Hormone OR T3 OR Diiodotyrosine OR Calcitonin OR T4 OR triiodothyronine OR thyroxine OR FT3 OR FT4 OR Free triiodothyronine OR free thyroxine)
#3	#1 AND #2
**Cochrane Library: 42 results retrieved from interception to January,08,2025**	
#1	(Bisphenol A) OR (BPA) OR (Bisphenol S) OR (BPS) OR (Bisphenol F) OR (BPF) OR (Bisphenol AF) OR (BPAF) OR (Bisphenol AP) OR (BPAP) OR (BPB)
#2	(Thyroid) OR (Thyroid Problem) OR (Thyroid Disease) OR (Thyroid Disorder) OR (Thyroid Dysfunction) OR (Thyroid Hormone) OR (T3) OR (Diiodotyrosine) OR (Calcitonin) OR (T4) OR (triiodothyronine) OR (thyroxine) OR (FT3) OR (FT4) OR (Free triiodothyronine) OR (free thyroxine)
#3	#1 AND #2

Table S2. PRISMA Checklist 2020.

		**TITLE**	
**Title**	**1**	Identify the report as a systematic review.	1
		**ABSTRACT**	
Abstract	2	See the PRISMA 2020 for Abstracts checklist.	2
		**INTRODUCTION**	
Rationale	3	Describe the rationale for the review in the context of existing knowledge.	3,4
Objectives	4	Provide an explicit statement of the objective(s) or question(s) the review addresses.	4
		**METHODS**	
Eligibility criteria	5	Specify the inclusion and exclusion criteria for the review and how studies were grouped for the syntheses.	5
Information sources	6	Specify all databases, registers, websites, organisations, reference lists and other sources searched or consulted to identify studies. Specify the date when each source was last searched or consulted.	4
Search strategy	7	Present the full search strategies for all databases, registers and websites, including any filters and limits used.	S1
Selection process	8	Specify the methods used to decide whether a study met the inclusion criteria of the review, including how many reviewers screened each record and each report retrieved, whether they worked independently, and if applicable, details of automation tools used in the process.	4
Data collection process	9	Specify the methods used to collect data from reports, including how many reviewers collected data from each report, whether they worked independently, any processes for obtaining or confirming data from study investigators, and if applicable, details of automation tools used in the process.	5
Data items	10a	List and define all outcomes for which data were sought. Specify whether all results that were compatible with each outcome domain in each study were sought (e.g. for all measures, time points, analyses), and if not, the methods used to decide which results to collect.	4,7,8
	10b	List and define all other variables for which data were sought (e.g. participant and intervention characteristics, funding sources). Describe any assumptions made about any missing or unclear information.	4,,8,9
Study risk of bias assessment	11	Specify the methods used to assess risk of bias in the included studies, including details of the tool(s) used, how many reviewers assessed each study and whether they worked independently, and if applicable, details of automation tools used in the process.	4,6
Effect measures	12	Specify for each outcome the effect measure(s) (e.g. risk ratio, mean difference) used in the synthesis or presentation of results.	6
Synthesis methods	13a	Describe the processes used to decide which studies were eligible for each synthesis (e.g. tabulating the study intervention characteristics and comparing against the planned groups for each synthesis (item #5)).	4
	13b	Describe any methods required to prepare the data for presentation or synthesis, such as handling of missing summary statistics, or data conversions.	N/A
	13c	Describe any methods used to tabulate or visually display results of individual studies and syntheses.	5
	13d	Describe any methods used to synthesize results and provide a rationale for the choice(s). If meta-analysis was performed, describe the model(s), method(s) to identify the presence and extent of statistical heterogeneity, and software package(s) used.	5
	13e	Describe any methods used to explore possible causes of heterogeneity among study results (e.g. subgroup analysis, meta-regression).	5
	13f	Describe any sensitivity analyses conducted to assess robustness of the synthesized results.	5
Reporting bias assessment	14	Describe any methods used to assess risk of bias due to missing results in a synthesis (arising from reporting biases).	5
Certainty assessment	15	Describe any methods used to assess certainty (or confidence) in the body of evidence for an outcome.	5
		**RESULTS**	
Study selection	16a	Describe the results of the search and selection process, from the number of records identified in the search to the number of studies included in the review, ideally using a flow diagram.	5
	16b	Cite studies that might appear to meet the inclusion criteria, but which were excluded, and explain why they were excluded.	5
Study characteristics	17	Cite each included study and present its characteristics.	7,8 (Table1)
Risk of bias in studies	18	Present assessments of risk of bias for each included study.	11, S1

Results of individual studies	19	For all outcomes, present, for each study: (a) summary statistics for each group (where appropriate) and (b) an effect estimates and its precision (e.g. confidence/credible interval), ideally using structured tables or plots.	10-14
	20a	For each synthesis, briefly summarise the characteristics and risk of bias among contributing studies.	10-14
Results of syntheses	20b	Present results of all statistical syntheses conducted. If meta-analysis was done, present for each the summary estimate and its precision (e.g. confidence/credible interval) and measures of statistical heterogeneity. If comparing groups, describe the direction of the effect.	10-14
	20c	Present results of all investigations of possible causes of heterogeneity among study results.	10-14
	20d	Present results of all sensitivity analyses conducted to assess the robustness of the synthesized results.	Figure S11-S16
Reporting biases	21	Present assessments of risk of bias due to missing results (arising from reporting biases) for each synthesis assessed.	Figure S6-S10
Certainty of evidence	22	Present assessments of certainty (or confidence) in the body of evidence for each outcome assessed.	Figure S17-S22
		**DISCUSSION**	
Discussion	23a	Provide a general interpretation of the results in the context of other evidence.	17
	23b	Discuss any limitations of the evidence included in the review.	19
	23c	Discuss any limitations of the review processes used.	19
	23d	Discuss implications of the results for practice, policy, and future research.	19
		**OTHER INFORMATION**	
Registration and protocol	24a	Provide registration information for the review, including register name and registration number, or state that the review was not registered.	22
	24b	Indicate where the review protocol can be accessed, or state that a protocol was not prepared.	22
	24c	Describe and explain any amendments to information provided at registration or in the protocol.	22
Support	25	Describe sources of financial or non-financial support for the review, and the role of the funders or sponsors in the review.	17
Competing interests	26	Declare any competing interests of review authors.	17
Availability of data, code, and other materials	27	Report which of the following are publicly available and where they can be found: template data collection forms; data extracted from included studies; data used for all analyses; analytic code; any other materials used in the review.	17

From: For more information, visit: http://www.prisma-statement.org/, accessed on: 23 September 2025

Table S3. PECO Statement.

P **Population**

- Adults and pregnant women exposed to bisphenols in Asia , Europe, and US

E **Exposure**

- bsphenol A and its alternatives (BPA, BPS, BPB, and BPF)

C **Comparator**

- No exposure to bisphenols

O **Outcome**

- Thyroid Hormones (TSH, FT3, FT4, T3, T4, TT3, and TT4) of adults and pregnant women

Table S4. Standardized units and corresponding conversion formulas for thyroid function analytes.

Analyte	Reported Unit(s)	Conversion Formula	Standard Unit
**TSH**	mIU/L	No Conversion	µIU/mL
**FT3**	pmol/L	1 pmol/L × 0.651	pg/mL
**FT3**	ng/dL	1 ng/dL ×10	pg/mL

**FT4**	pmol/L	1 pmol/L × 0.0777	ng/dL
**TT3**	nmol/L	1 nmol/L × 65.1	ng/dL
**TT3**	µg/dL	1 nmol/L × 1000	ng/dL
**TT4**	nmol/L	1 nmol/L × 0.0777	µg/dL
**TT4**	µg/mL	1 µg/mL ×10	µg/dL

Table S5. Detailed Newcastle-Ottawa Scale of each included cohort study.

**Study (First Author)**	**Study Design**	**Selection**				**Comparability**	**Outcome/Exposure**			
		**Representa-tiveness of exposed**	**Selection of non-exposed**	**Ascertain-ment of exposure**	**Outcome, not initial present**	**Based on design and analysis**	**Assessment of outcome**	**Long follow-up for outcome**	**Adequate cohort follow-up**	**Score**
**Aker et al. 2019**	**Cohort**	1	1	1	1	2	1	0	1	08
**Derakhshan et al. 2019**	Cohort	1	1	1	1	2	1	1	0	08
**Derakhshan et al. 2020**	Cohort	1	1	1	1	2	1	1	0	08
**Aker et al. 2018**	Cohort	1	1	1	1	2	1	0	0	07
**Chevrier et al. 2023**	Cohort	1	1	1	1	2	1	0	1	08
**Geens et al. 2015**	Cohort	1	1	1	1	2	1	1	1	09
**Wang et al. 2020**	Cohort	1	1	1	1	2	1	1	0	08
**Aker et al. 2016**	Cohort	1	1	1	1	2	1	0	1	08
**Aung et al. 2017**	Cohort	1	1	1	1	2	1	0	1	08
**Ryva et al. 2024**	Cohort	1	1	1	1	1	1	0	1	08
**Study (First Author)**	**Study Design**	**Representativeness of the sample**	**sample size**	**Non-respondents**	**Ascertainment of Exposure**	**Based on design and analysis**	**Assessment of outcome**	**Statistical test**		
**Kwon et al. 2020**	Cross-Sectional	1	1	1	1	2	2	1	09	
**Yue et al. 2023**	Cross-Sectional	0	0	1	1	2	2	1	07	
**Park et al. 2017**	Cross-Sectional	1	1	1	1	2	1	1	09	
**Meeker & Ferguson. 2011**	Cross-Sectional	1	1	1	1	2	1	1	09	
**Wang et al. 2013**	Cross-Sectional	1	1	1	1	2	1	1	09	
**Cheng et al. 2023**	Cross-Sectional	1	1	1	1	2	1	1	09	
**Gao et al. 2024**	Cross-Sectional	1	1	1	1	2	2	1	09	
**Hu et al. 2023**	Cross-Sectional	1	0	1	1	1	1	1	07	

1) Was follow-up long enough for outcomes to occur: 1, duration of follow-up >=3 years; 0 if duration of follow-up < 3 years.

2) Loss to follow-up rate: 1, complete follow-up or loss to follow-up rate <=20 %; 0, follow-up rate < 80% or no description of those lost.

Very Good Studies: 9-10 points; Good Studies: 7-9 points; Satisfactory Studies; 5-6 points; Unsatisfactory Studies: 0 to 4 points

Table S6. study characteristics (concentrations).

**Author_year**	**Exposure**	**Unit**	**Concentration**	**GM**	**LOD**	**Outcome-units**
**Aker et al. 2019**	**A, S, F**	ng/ml	6.18, 4.23, 2.09 (95th)	1.88, 0.54, 0.31	0.1, 0.2,0.2	TSH- uIU/mL, FT4- ng/dl, T3- ng/mL, T4- ug/L
**Derakhshan et al. 2019**	**A, S, F**	ng/ml	(0.34–9.36), (0.03–0.88), (0.03–7.88)	1.51, 0.08, 0.15 (M)	0.22, 0.03, 0.03	TSH- mIU/L, FT4-pmol/L, FT3-pmol/L, TT4-nmol/L, TT3-nmol/L

**Derakhshan et al. 2021**	**A, S, F**	ng/ml	<LOD-21.2, <LOD-1.69, N/A	1.47, 0.24 (M)	0.15, 0.05, 0.18	TSH-mIU/L, FT4-pmol/L, TT4-nmol/L
**Aker et al. 2018**	**S**	ng/ml	N/A	1.19 (SD 7.14)	(0.4)	TSH-µIU/mL, FT4- ng/dl, T4-ng/mL, T3-µg/dL
**Kwon et al. 2020**	**A**	ng/ml	2.98 (75th), N/A (95th)	1.48(SE 0.04)	0.15	TSH-uIU/mL, T4-ug/dl, T3-ng/dl
**Chevrier et al. 2013**	**A**	ug/g	1.9 (75th), N/A (95th)	1.3 (SD 2.0)	0.4 ug/l	TSH-mIU/L, FT4-ng/dL, TT4-µg/dL
**Geens et al. 2015**	**A**	ng/mL	5.1 (95th)	1.7 (M)	N/A	TSH-mIU/L, FT4-(pmol/L)
**Yue et al. 2023**	**A, S, F**	ng/ml	0.9, 0.17, 0.41 (90th) 0.57, 0.09, 0.15 (75th)	0.87±(SD4.13), 0.07±(SD 0.12), 0.23±(SD 0.78)	0.005, 0.005, 0.002	TSH-uIU/mL, FT4-ng/dl, FT3-ng/dl, TT4-ug/dl, TT3-ug/dl
**Wang et al. 2020**	**A**	ng/ml	95% CI 1.17–1.49	1.32	0.1	TSH-mIU/L, FT4-pmol/L)
**Aker et al. 2016**	**A**	ng/mL	N/A	N/A	N/A	TSH-uIU/mL, FT4- ng/dL, FT3-pg/mL
**Aung et al. 2017**	**A**	ng/ml	5.59	1.18 (SD 2.82)	18.9	TSH- µIU/mL, FT4-ng/dl, TT4-µg/dL, TT3-ng/mL
**Park et al. 2017**	**A**	ng/ml	8.314	1.126	0.15	TSH-µIU/mL, TT4-µg/dL, TT3-ng/dl
**Meeker & Ferguson. 2011**	**A**	ug/g	8.87	2.03	7.1	TSH- µIU/mL, FT4-ng/dl, FT3-pg/ml, TT4-µg/mL, TT3-ng/dL
**Wang et al. 2013**	**A**	ng/ml	IQR (0.47–1.43)	0.81 (M)	0.30	FT3-pmol/L, FT4-pmol/L, TSH-µIU/ml
**Cheng et al. 2023**	**A**	ng/ml	IQR (1.70 (0.90, 3.40)	1.72	0.4	FT4 (ng/dL), FT3 (pg/mL), TSH (mIU/L), TT4 (µg/dL), TT3 (ng/dL)
**Gao et al. 2024**	**A**	ng/ml	95th 10.47	N/A	0.4	T3 (ng/dL), T4 (µg/dL), FT3 (pg/mL)
**Hu et al. 2023**	**A**	ug/g	0.04-25.56	SD 1.29	0.08	FT3 (pmol/L), TT3 (nmol/L), FT4 (pmol/L), TT4 (nmol/L), TSH (uIU/mL)
**Ryva et al. 2024**	**A, S**	ng/ml	(0.52, 1.46), (0.29, 0.80)	0.85, 0.48	0.2, 0.1	FT4 ng/dL, TSH µIU/mL, TT4 µg/dL

Note: All units were standardized to the TSH=uIU/ml, FT3=pg/mL, FT4=ng/dl, TT3=ng/dL, TT4=ug/dL.

footnote 1: concentration: bisphenols concentrations; GM: geometric means; SD: standard deviation; SE: standard error; M: median; LOD: limit of detection; are these values given in the table respectively to the exposure

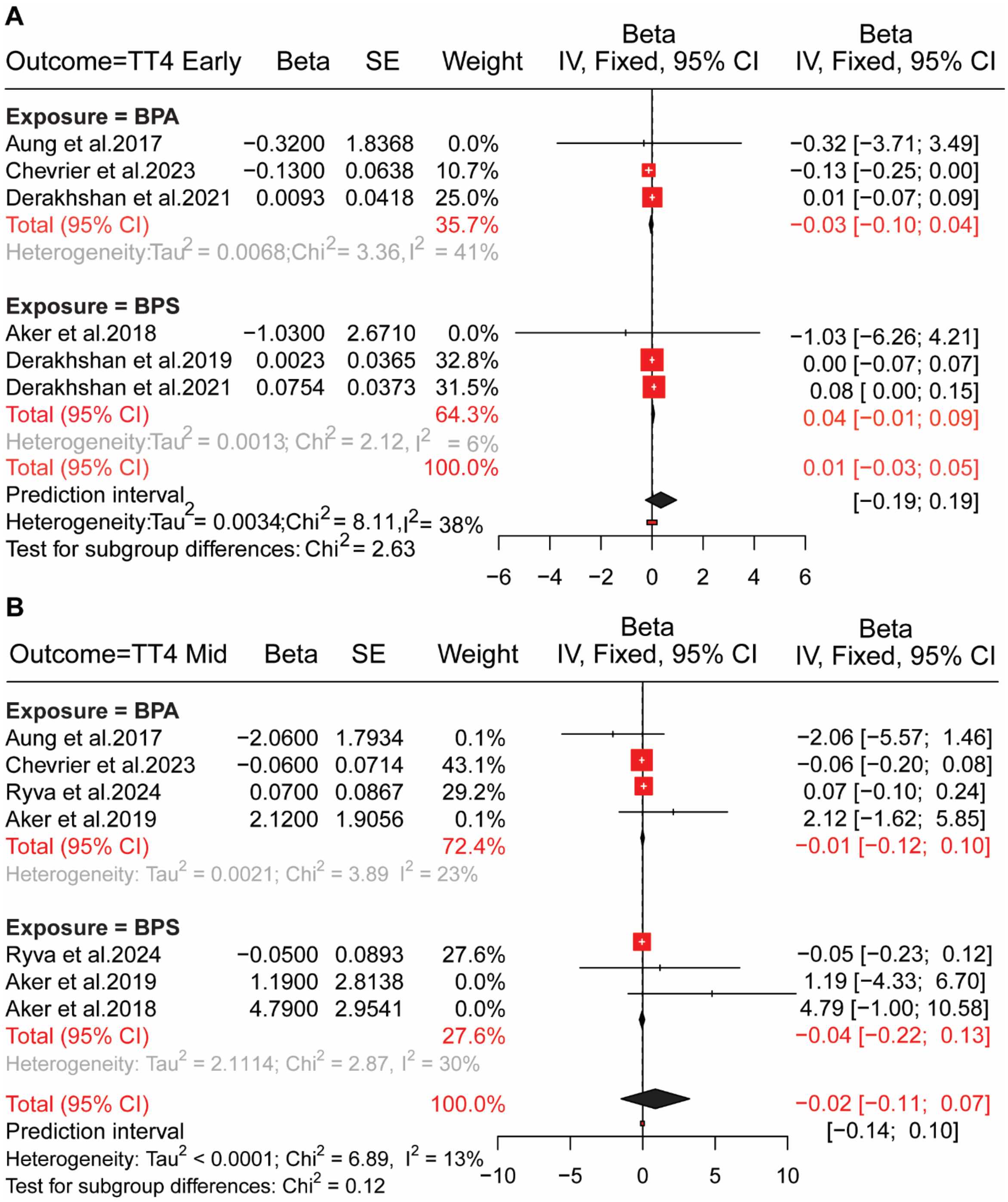

Footnote: TT4-Total thyroxine, early pregnancy-1st trimester of pregnant women, mid pregnancy-2nd trimester of pregnant women, BPA-Bisphenol A, BPS-Bisphenol S

Figure S1. Association between bisphenols exposure to total thyroxine (TT4) in pregnant women.

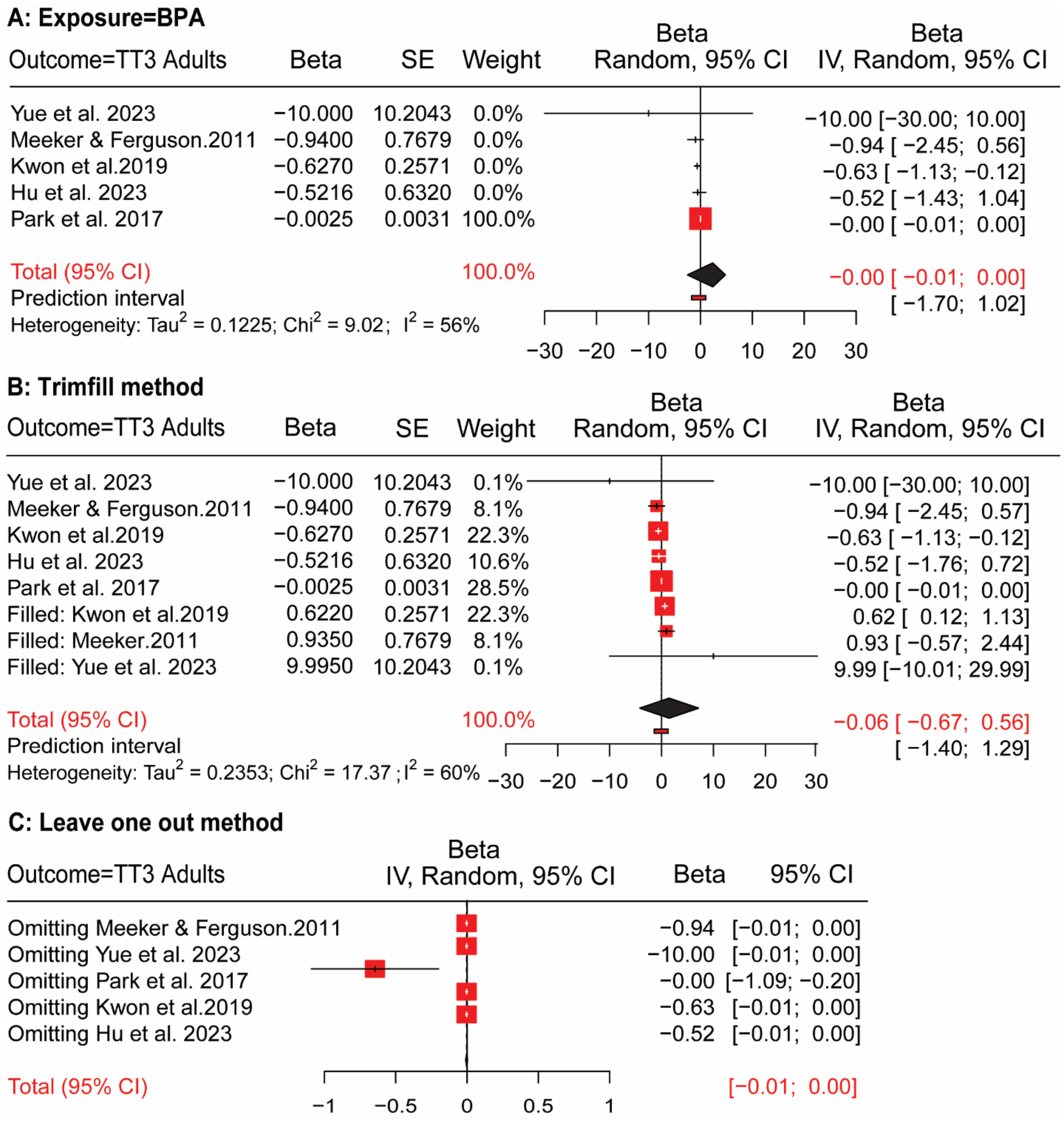

Figure S1: Association between bisphenols exposure to total triiodothyronine in adults and trim fill results

Footnote: TT3-Total triiodothyronine, A-Adults: a group including adult boys and girls without pregnancy, B & C are sensitivity analysis

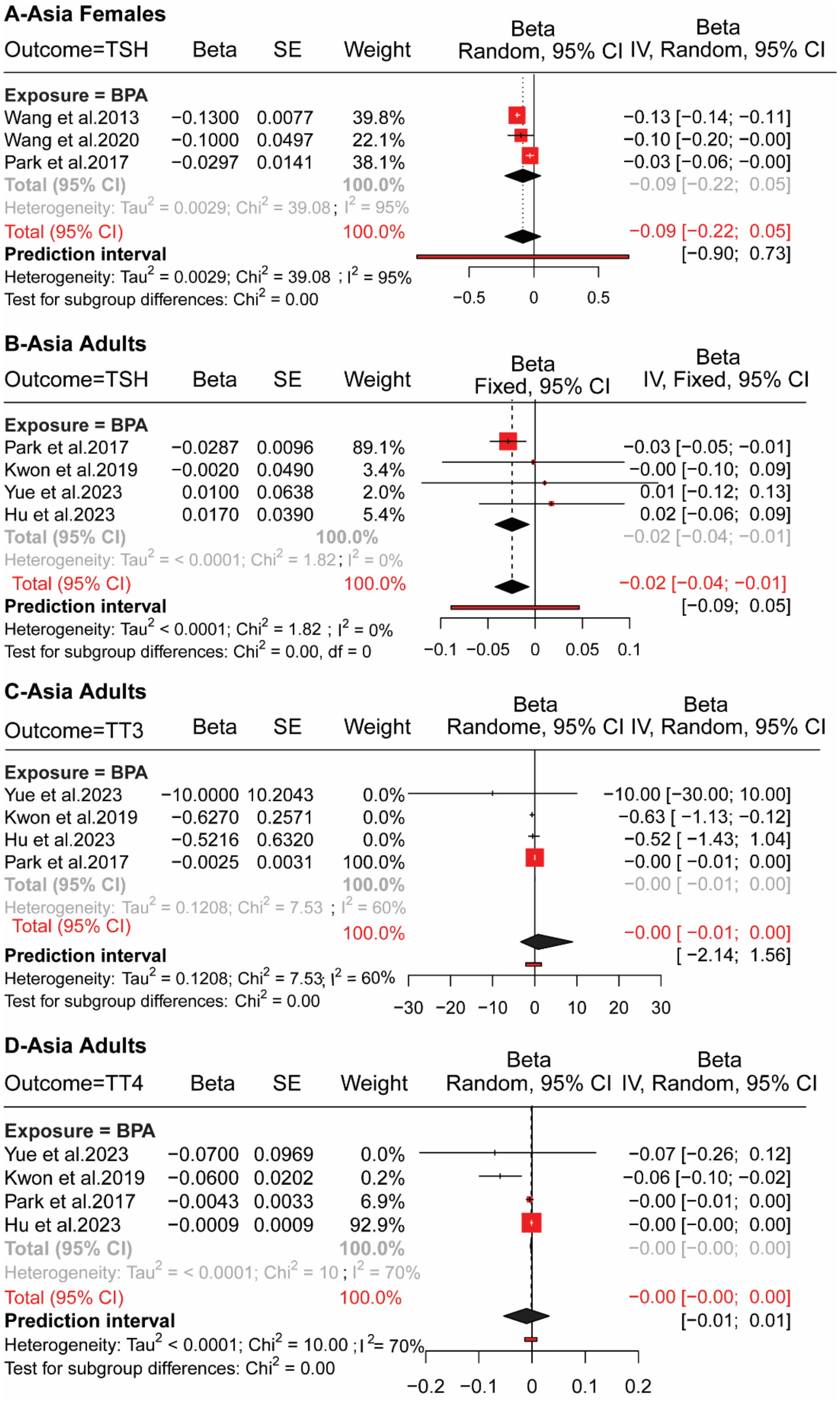

Figure S2: Association between bisphenols exposure to HPT axis hormones in Asian adults

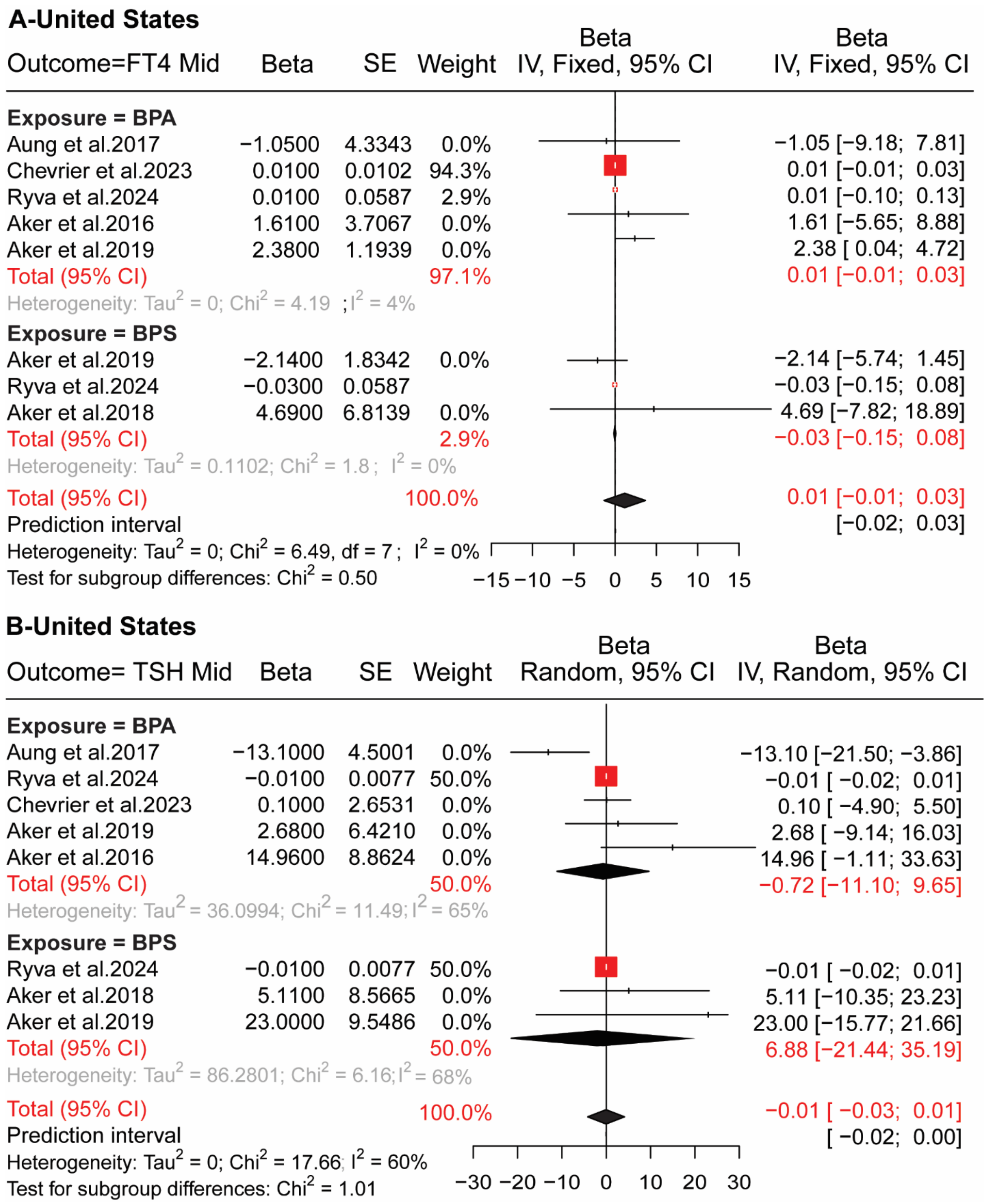

Figure S3: Association between bisphenols exposure to HPT axis hormones in US pregnant women

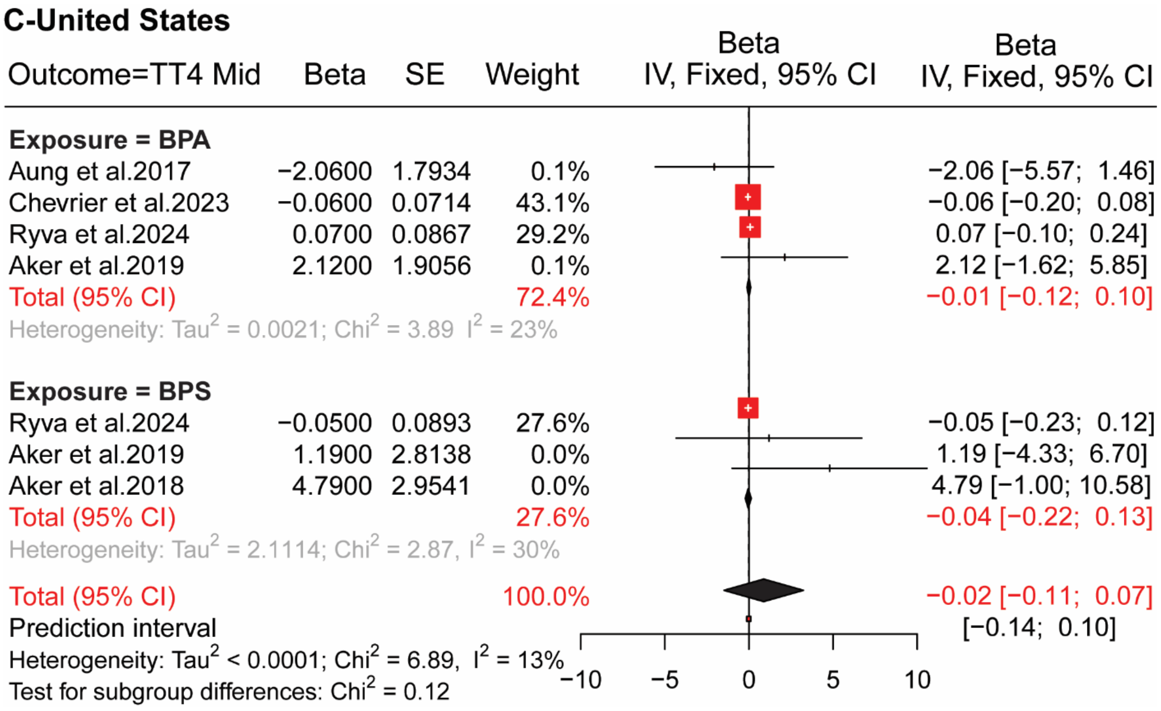

Figure S4: Association between bisphenols exposure to total thyroxine in mid pregnancy

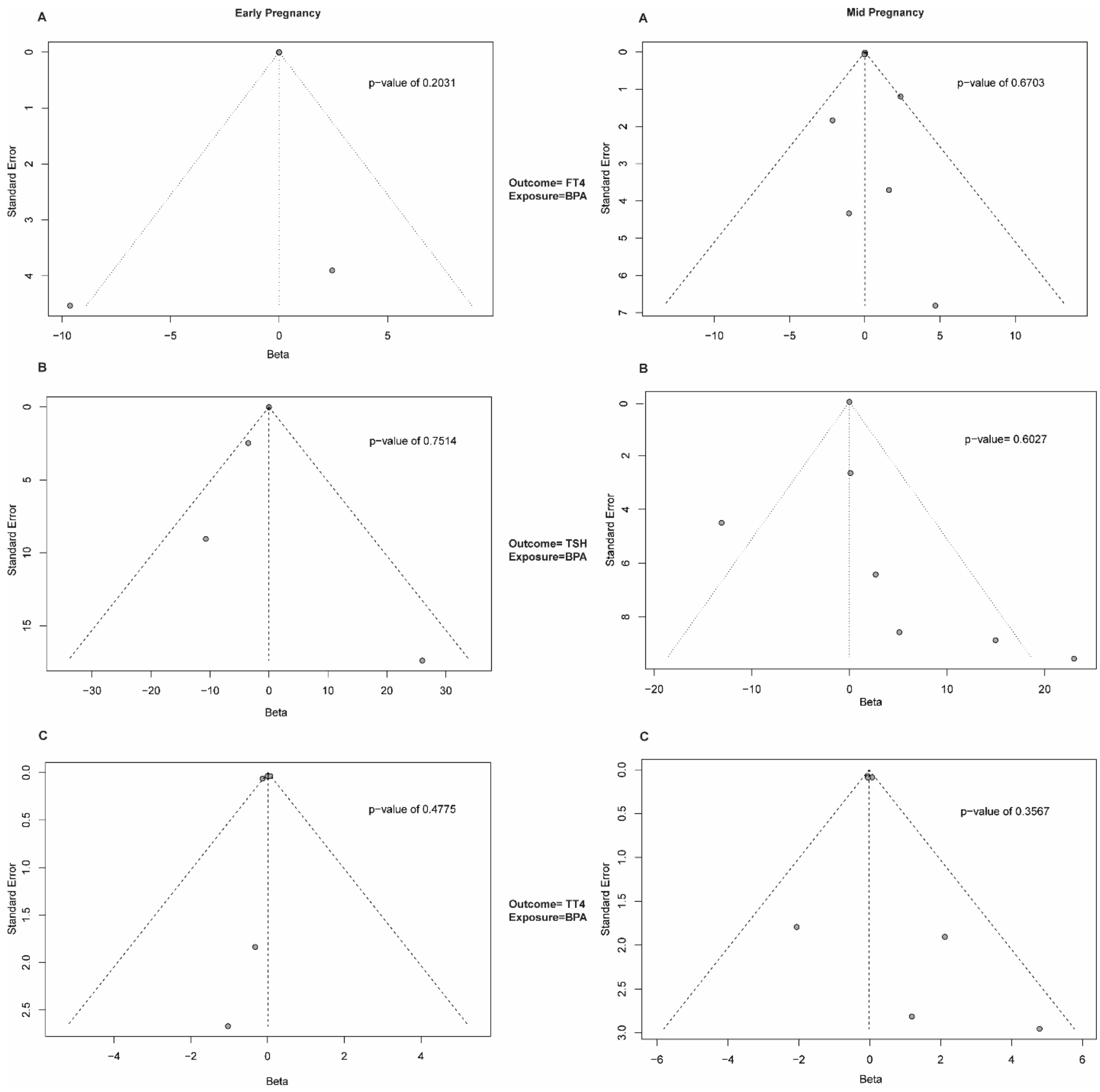

Figure S5: Publication bias for pregnant women

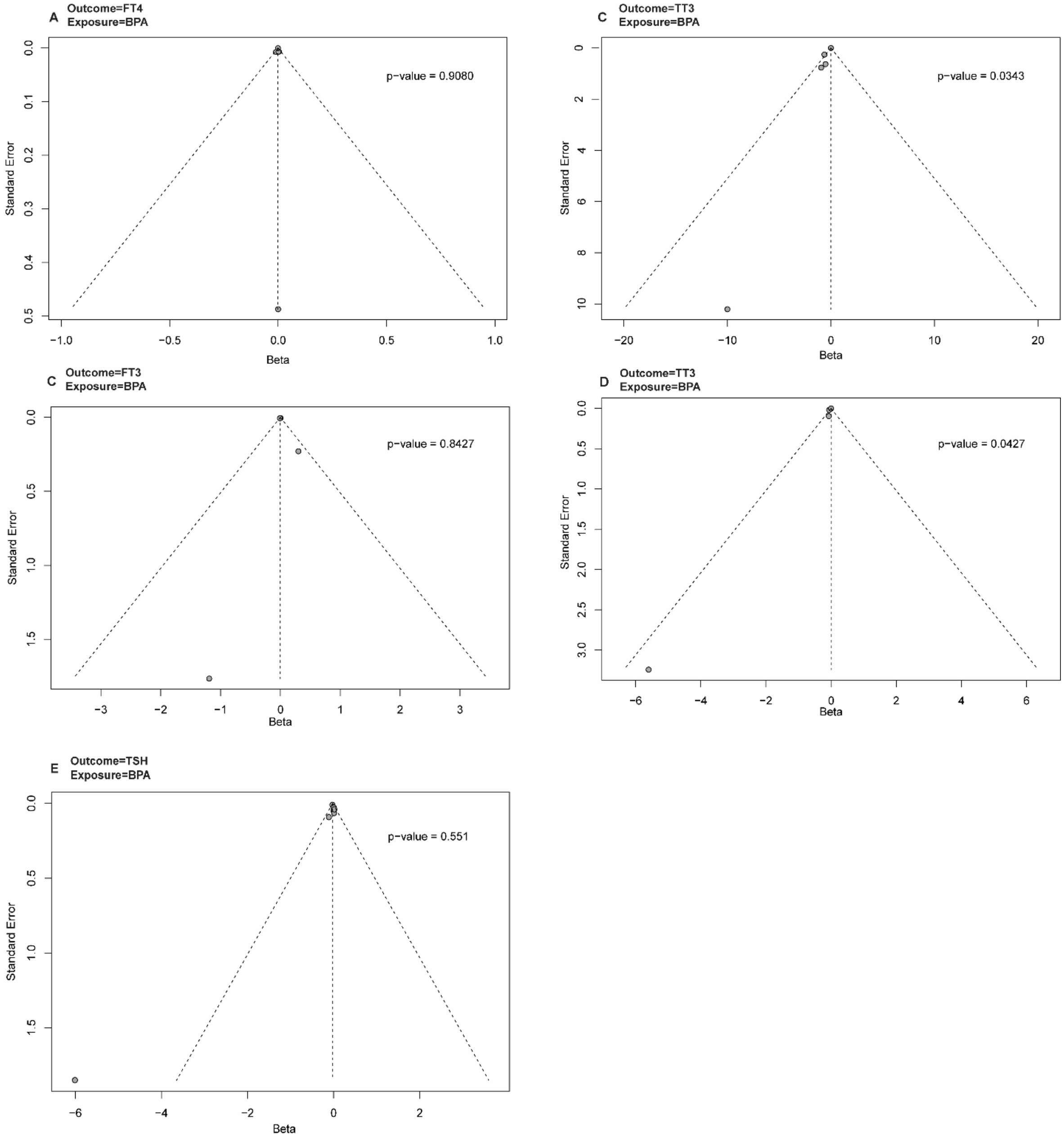

Figure S6: Publication Bias for adult studies

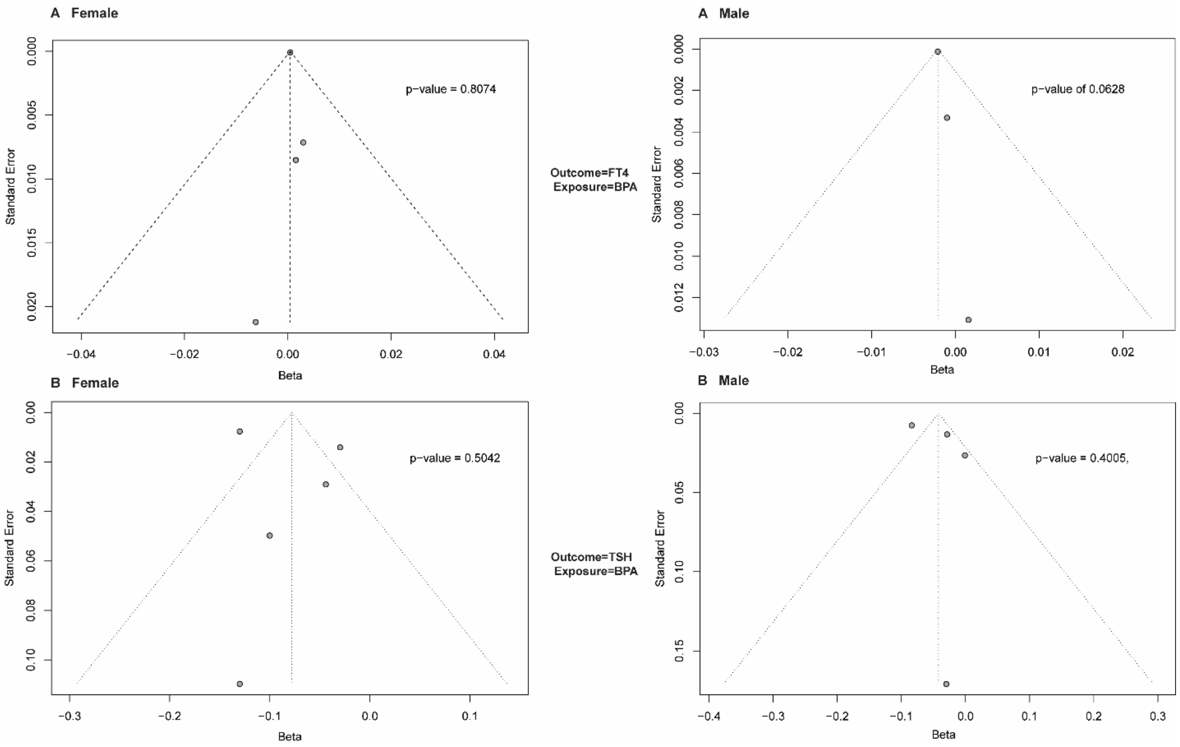

Figure S7: Publication Bias for female and male gender

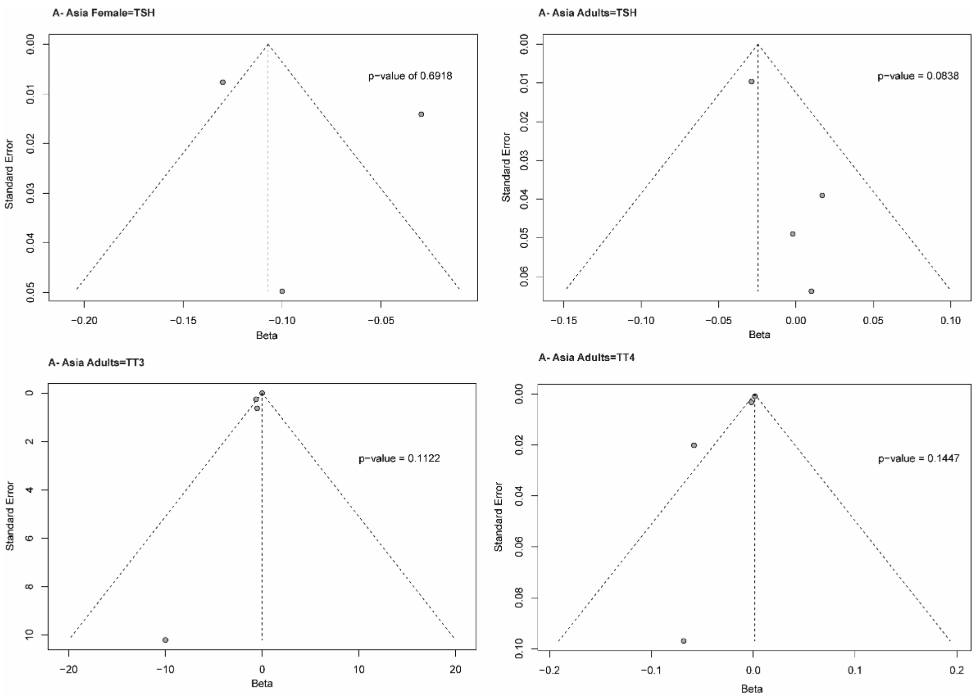

Figure S8: publication Bias for Asia region

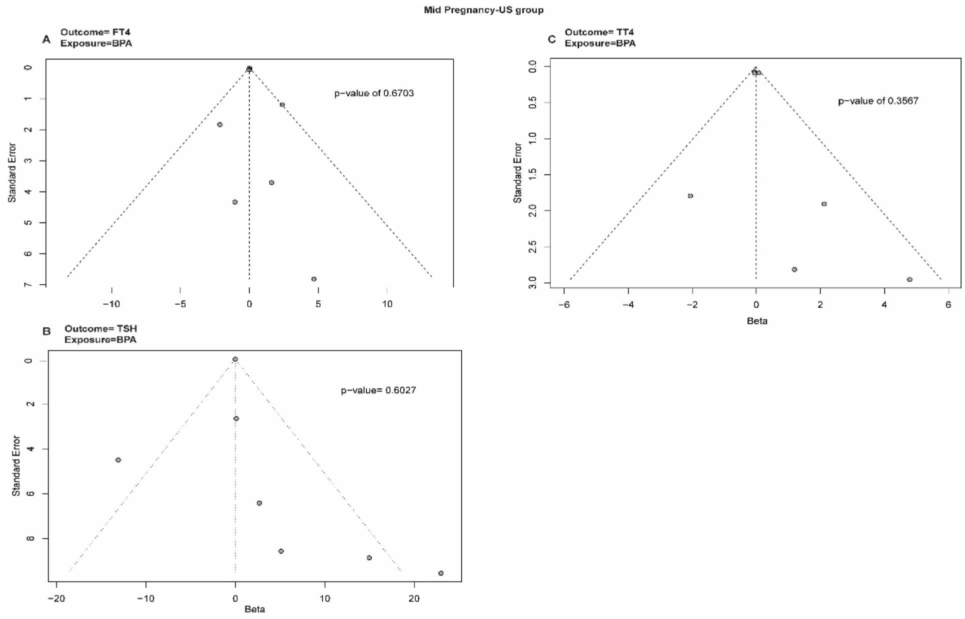

Figure S9: Publication bias for US region

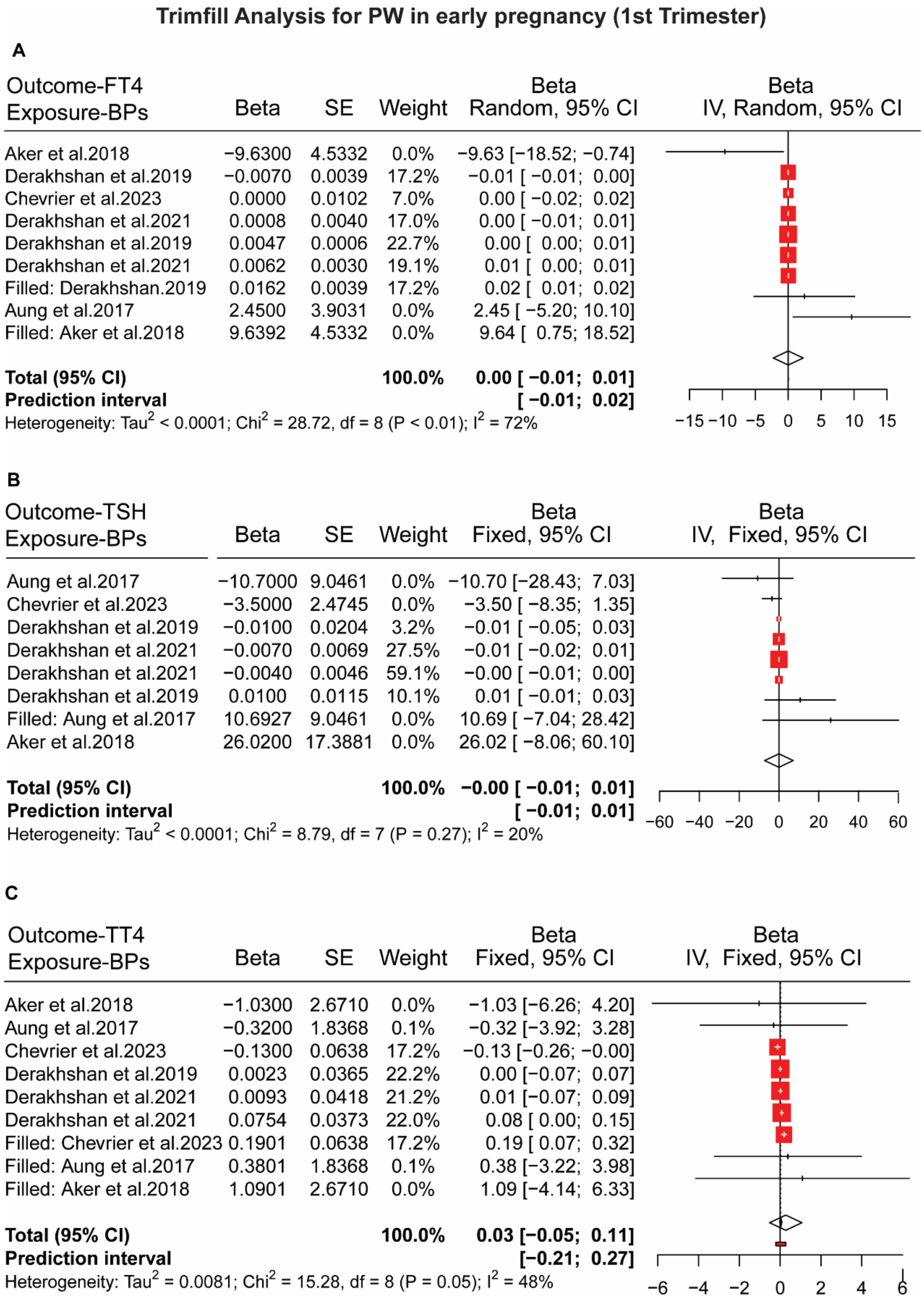

Figure S10: trim fill analysis results in early pregnancy

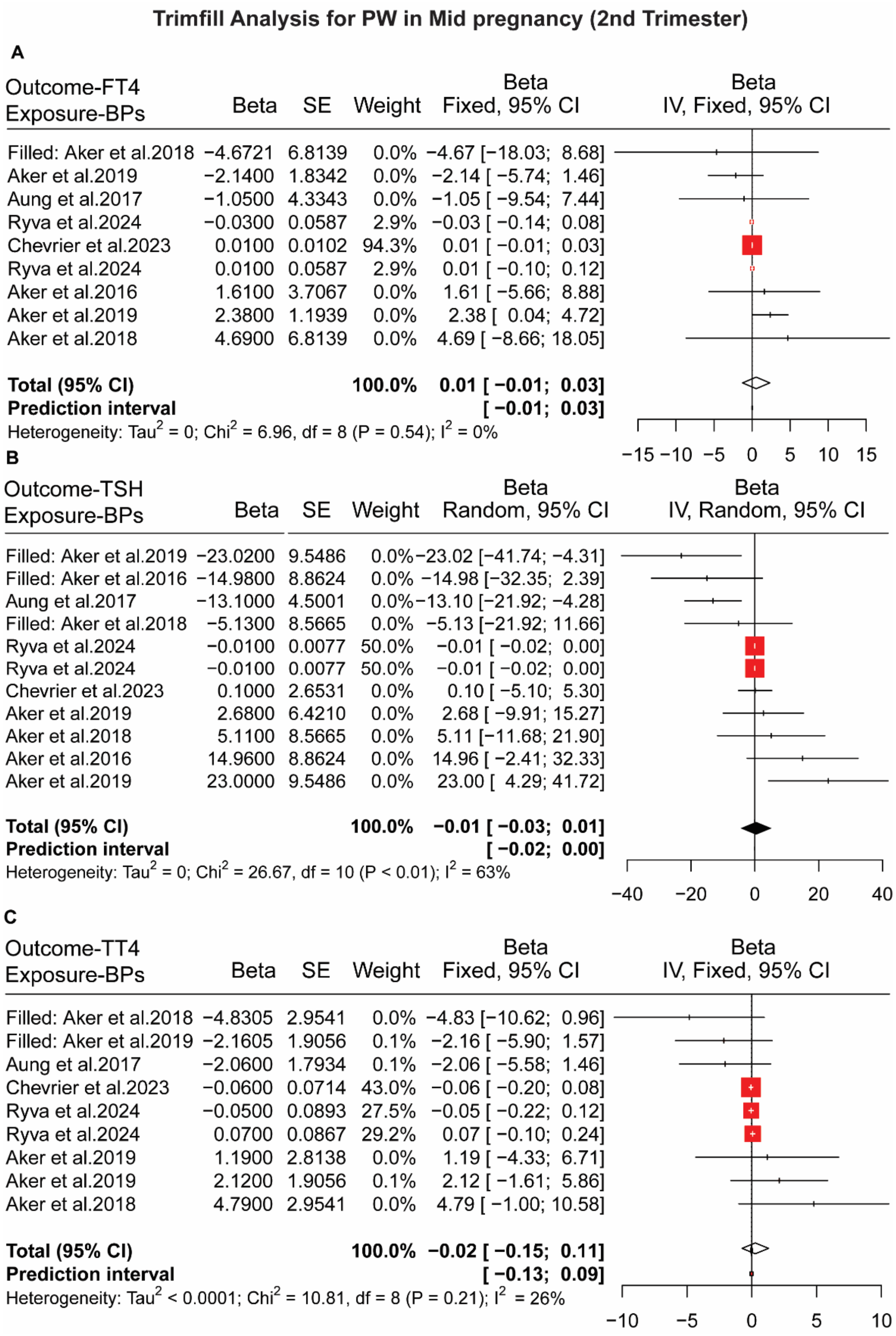

Figure S11: trim fill analysis results in mid pregnancy

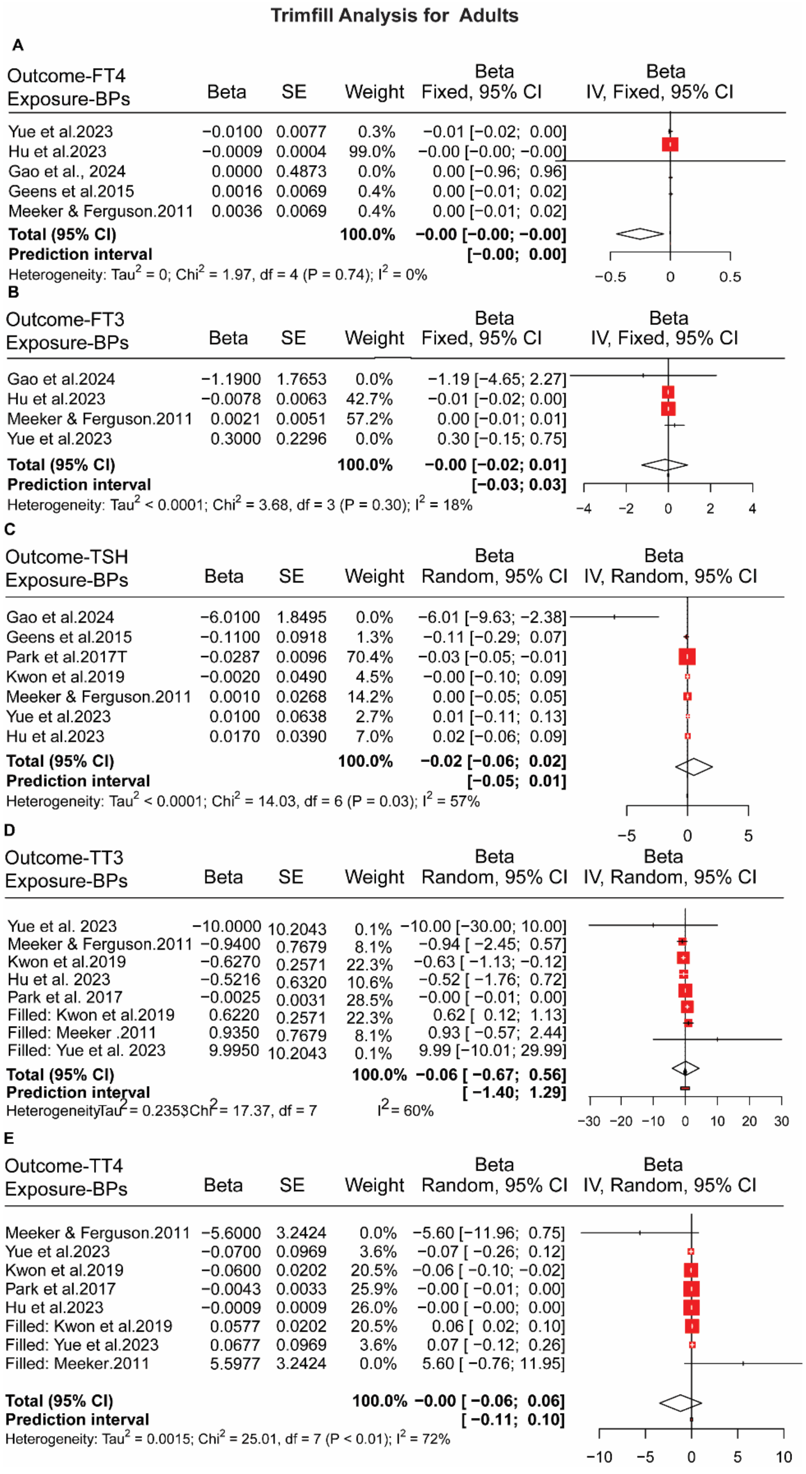

Figure S12: trim fill analysis results in adults

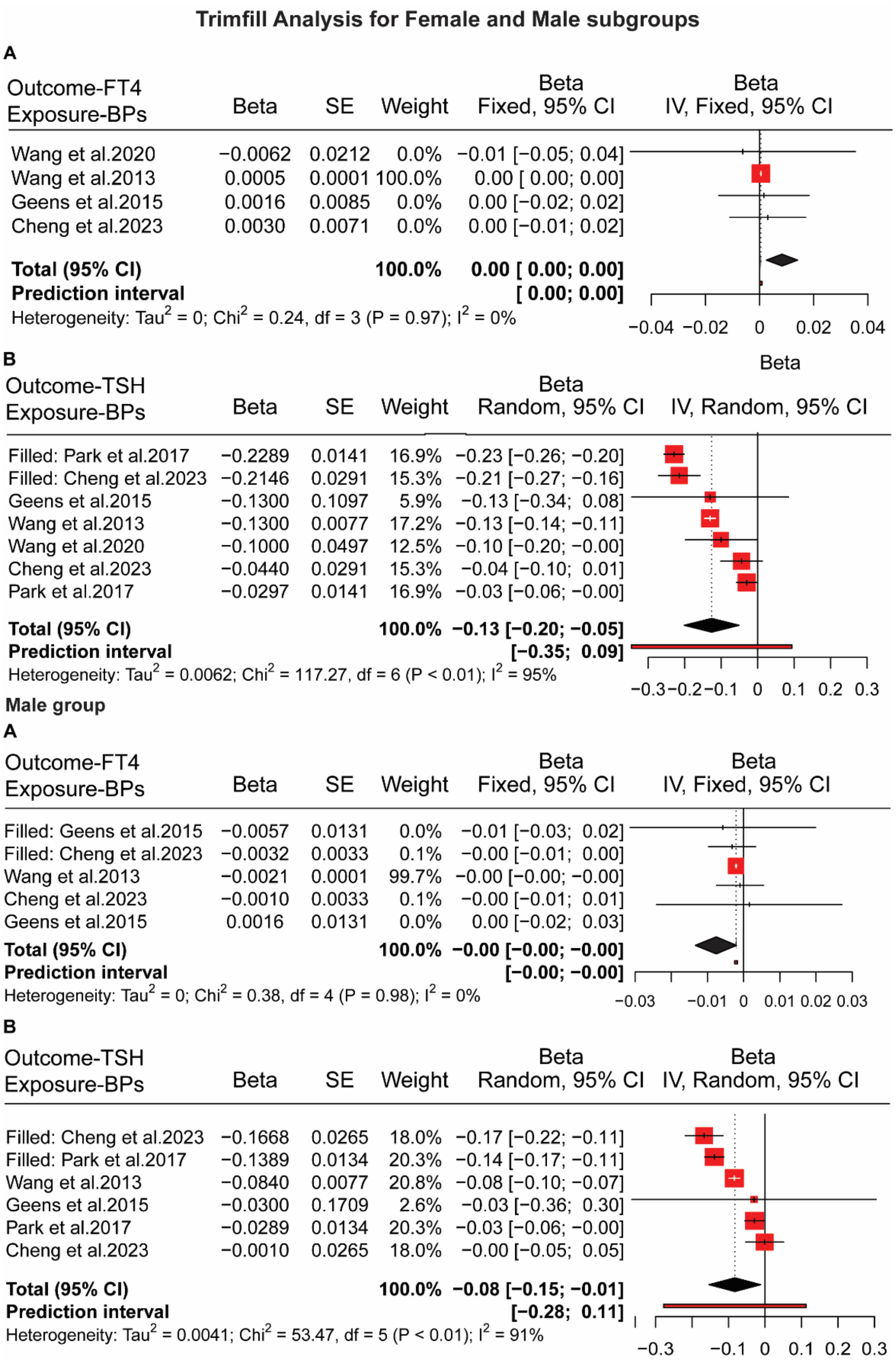

Figure S13: trim fill analysis results in both gender

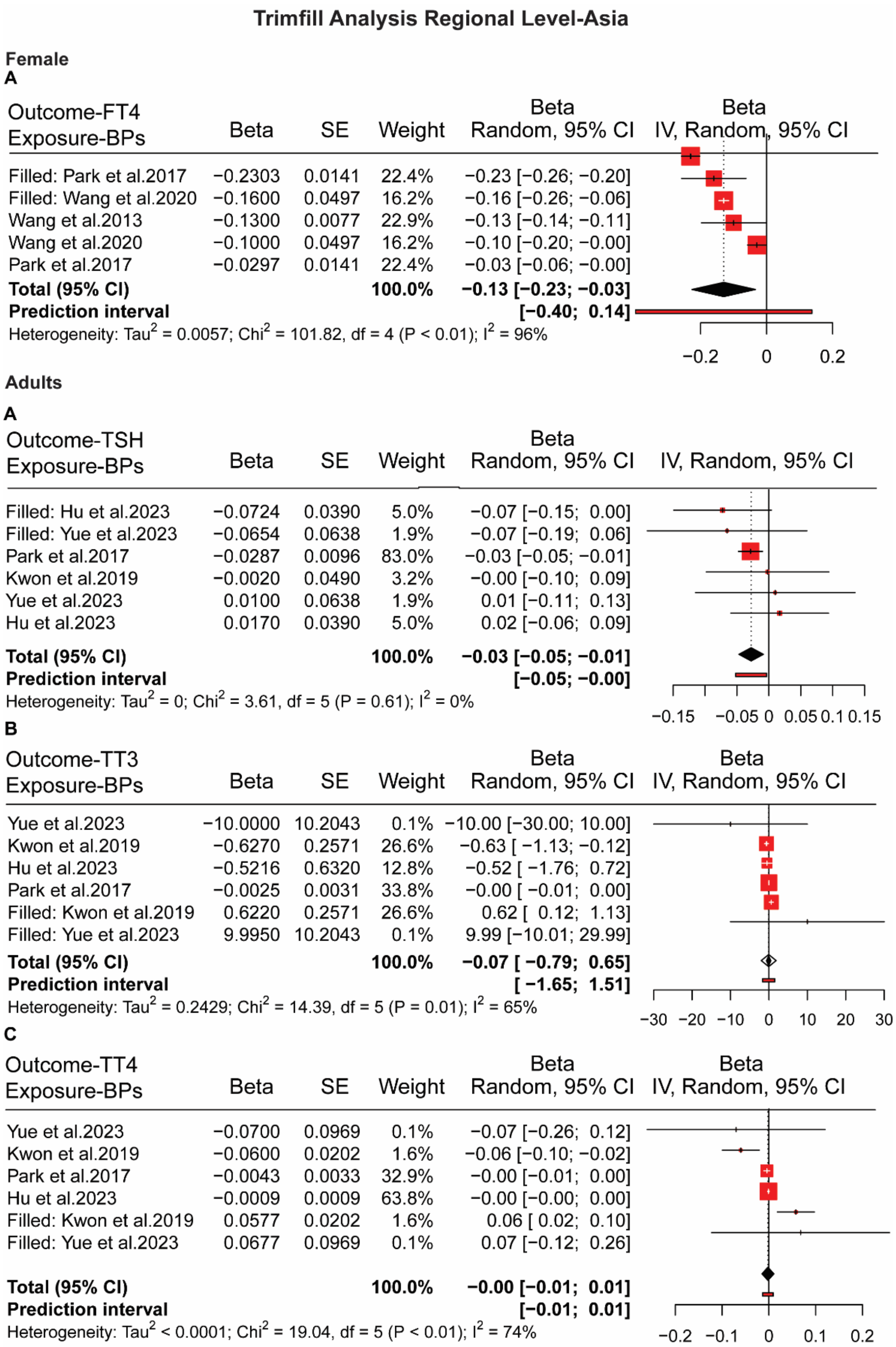

Figure S14: trim fill analysis results for Asia region

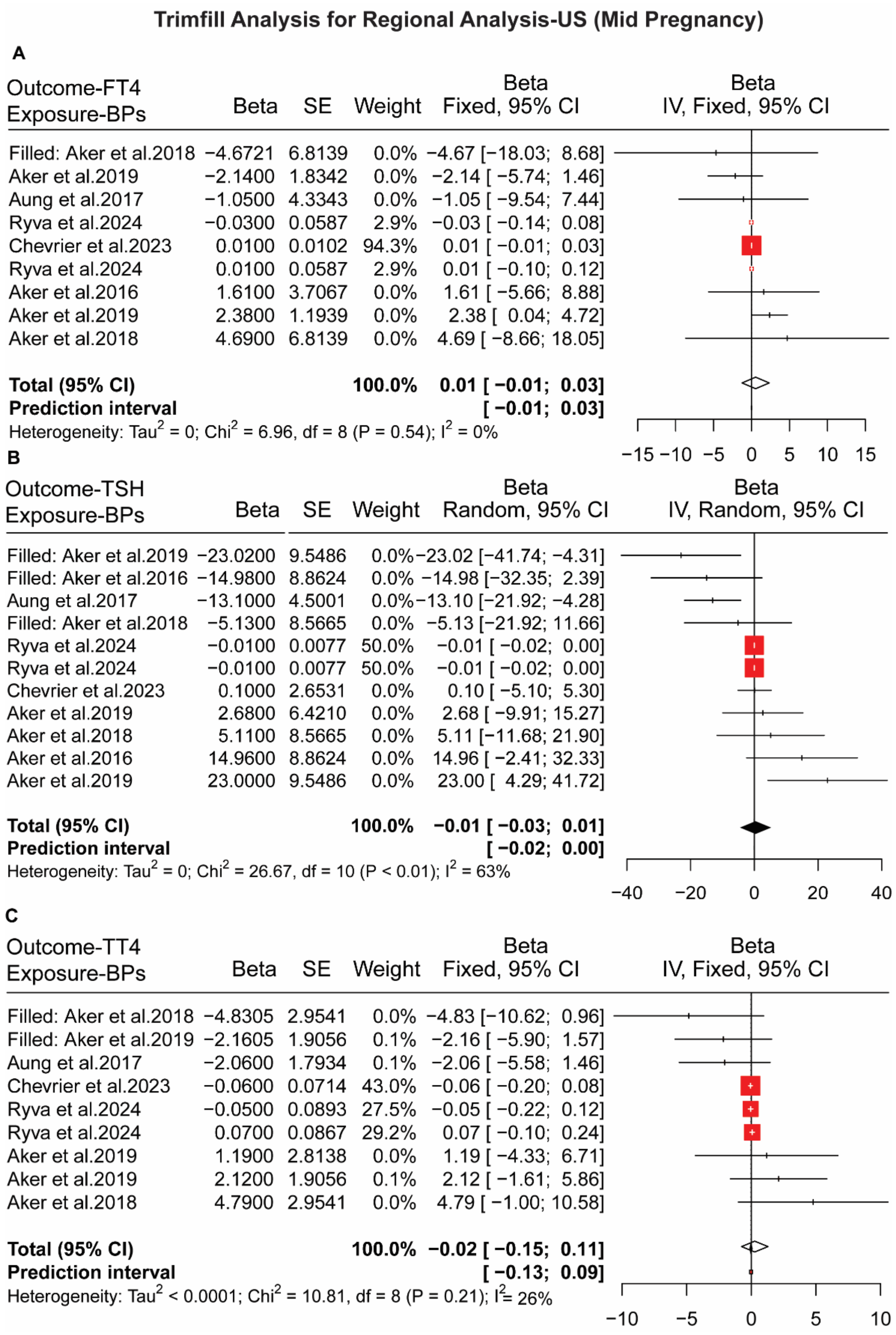

Figure S15: trim fill analysis results for US region

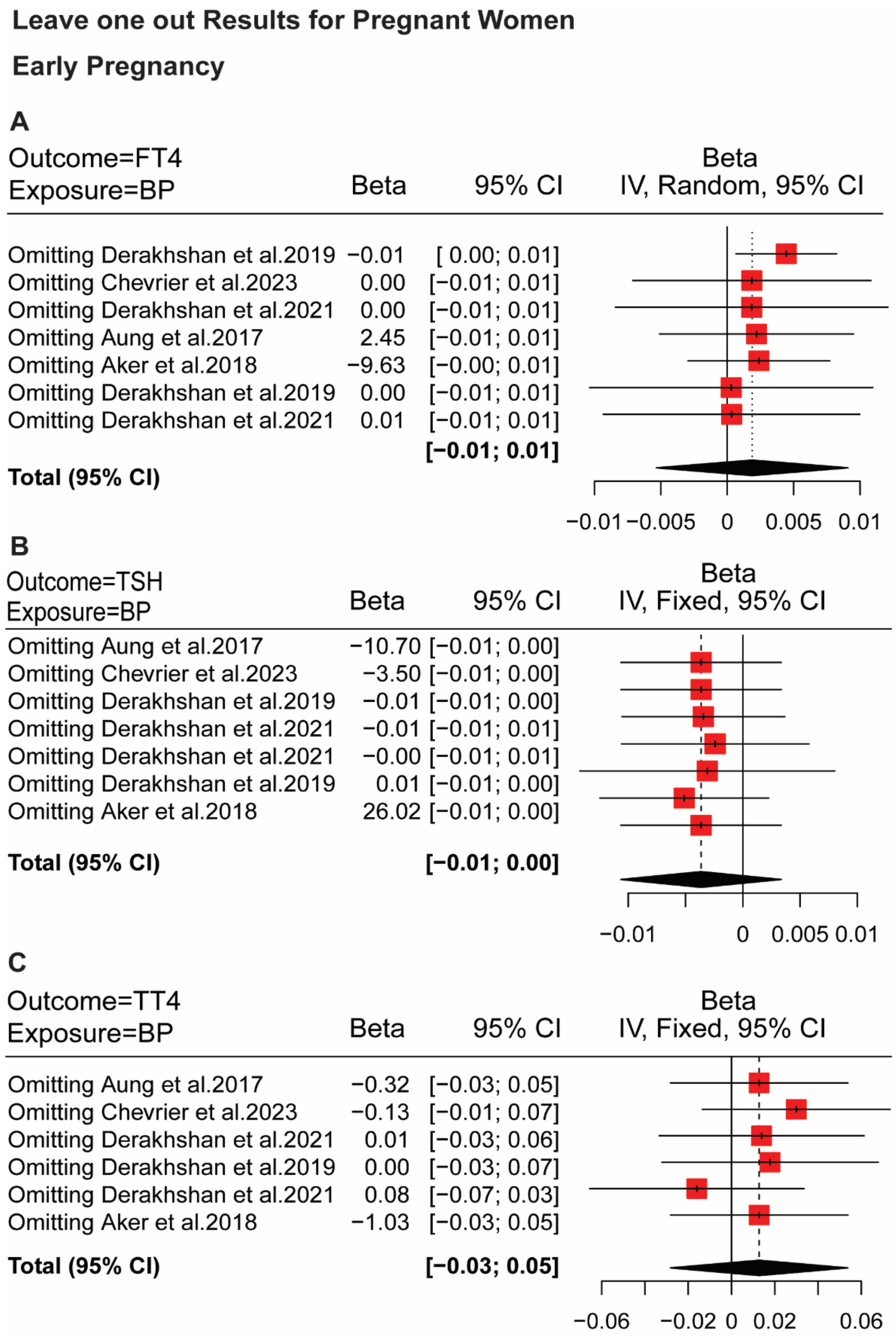

Figure S16: leave one out method results for pregnant women in early pregnancy

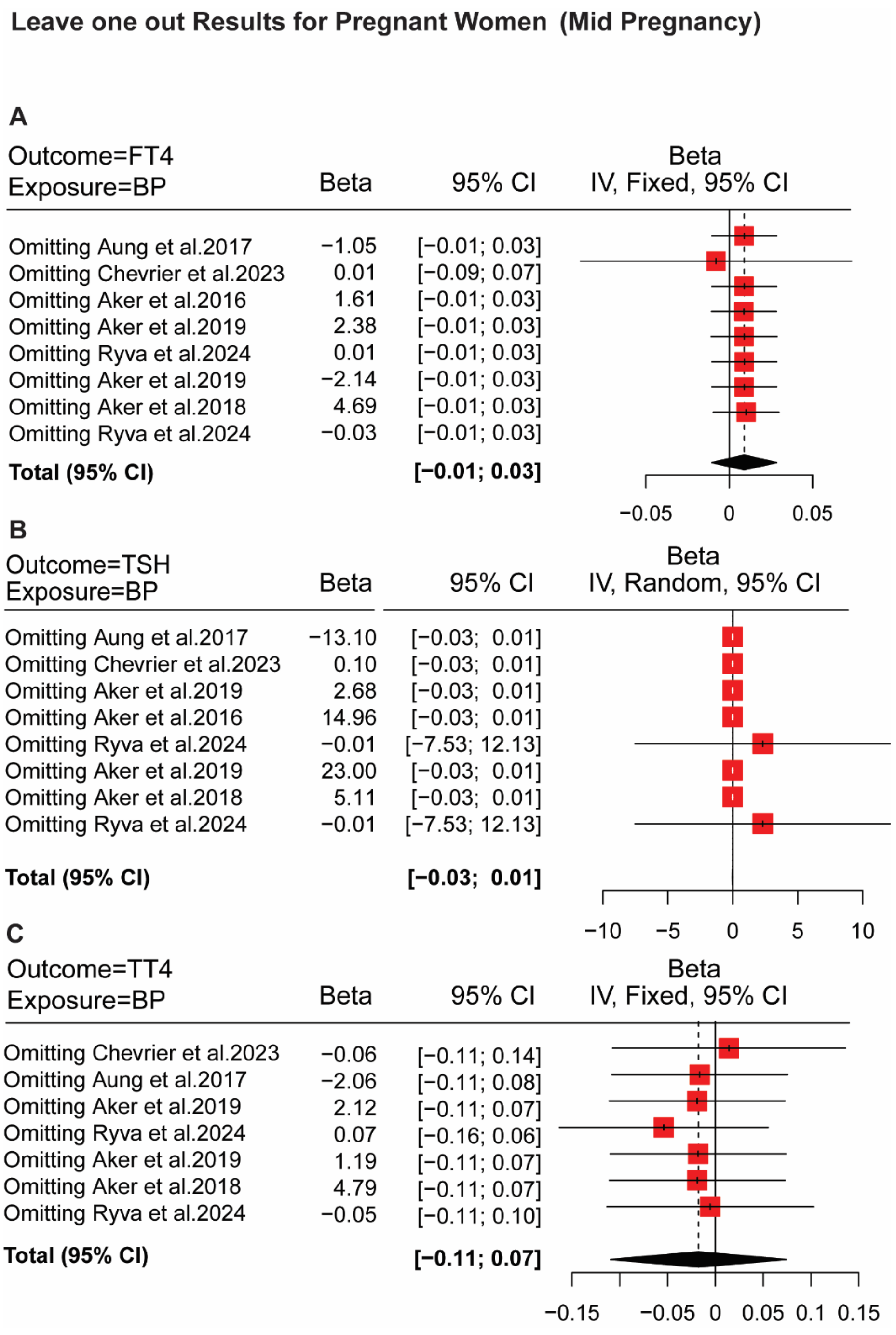

Figure S17: leave one out method results for pregnant women in early pregnancy

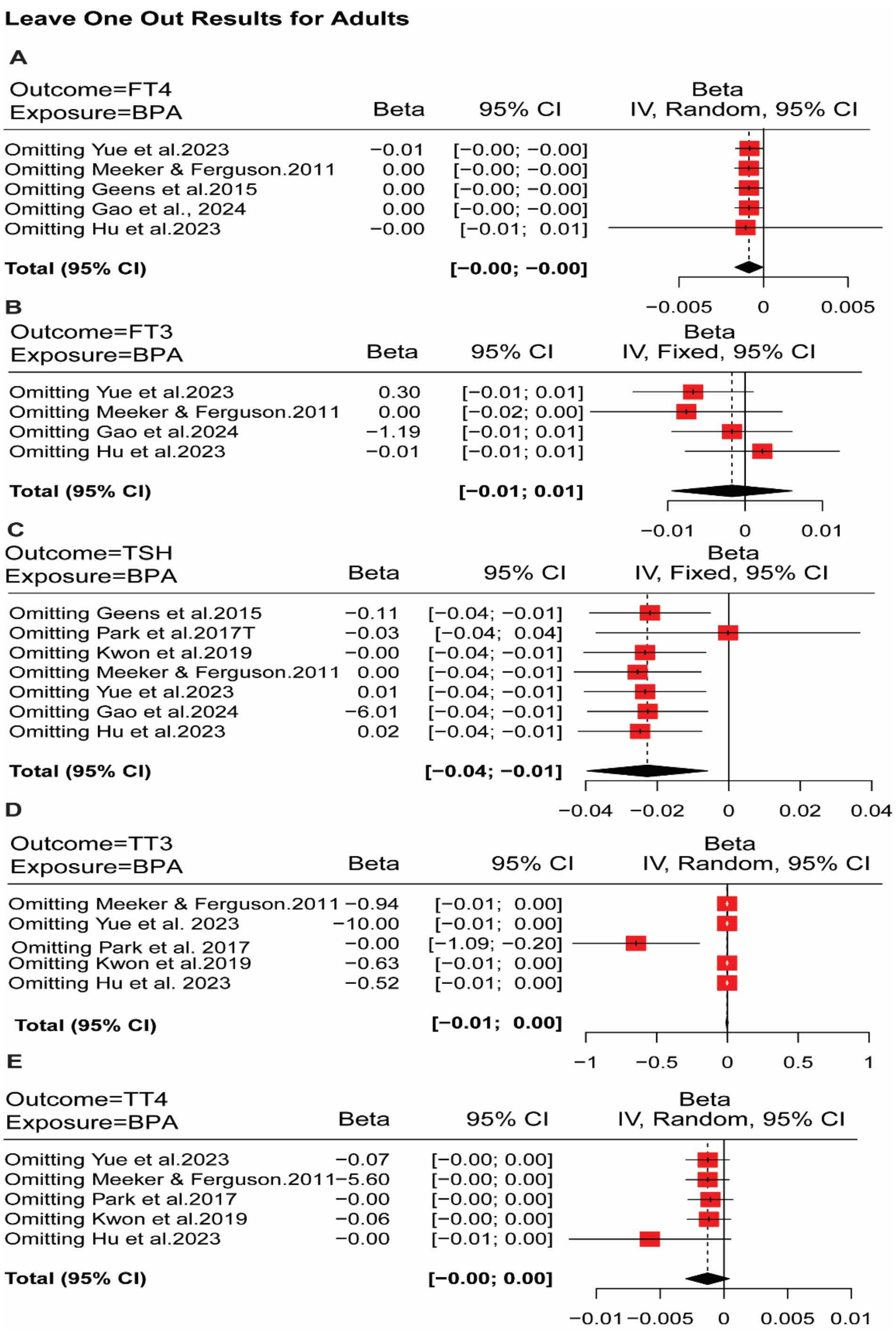

Figure S18: leave one out method results for adults

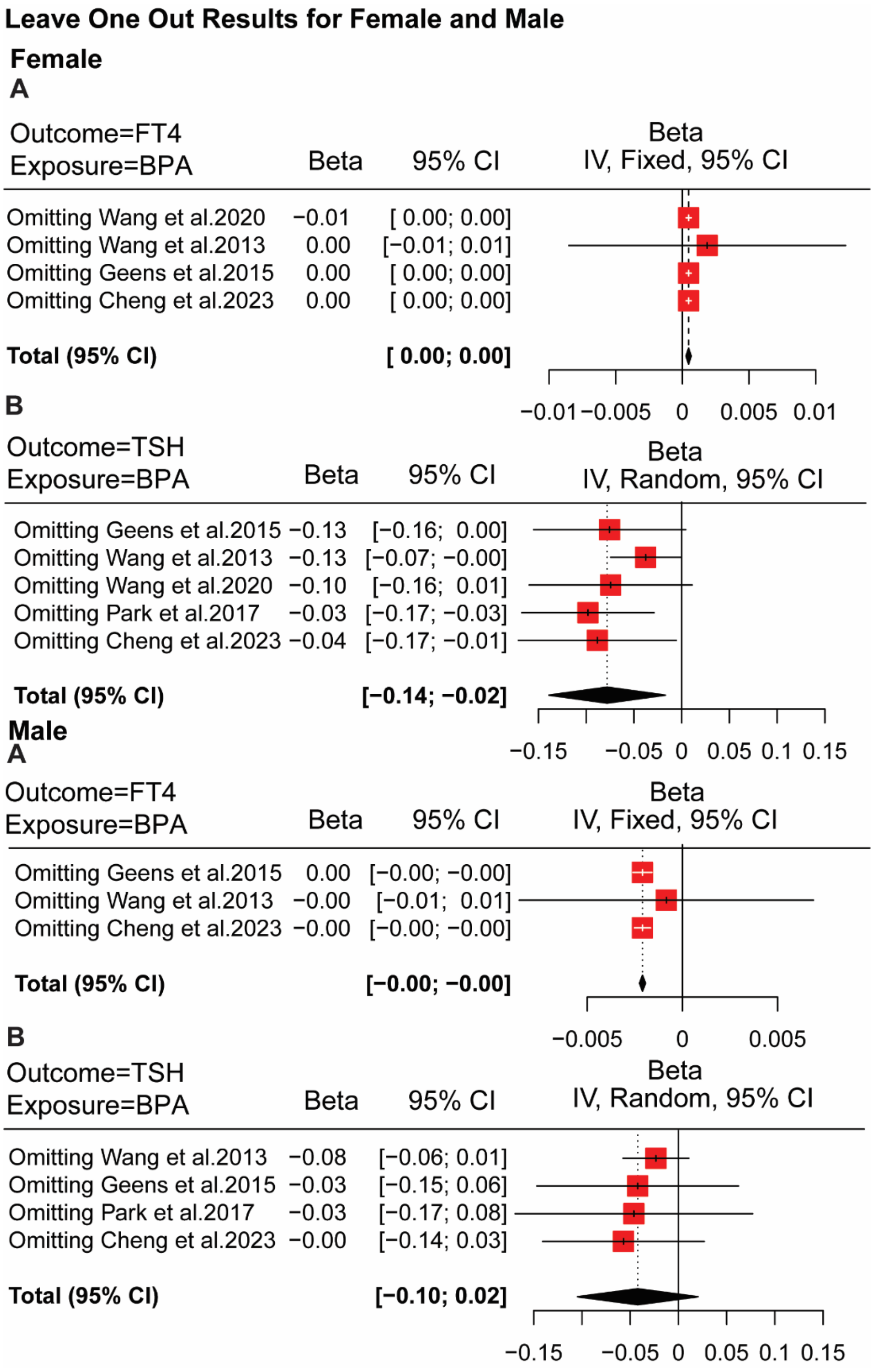

Figure S19: leave one out method results for female and male subgroups

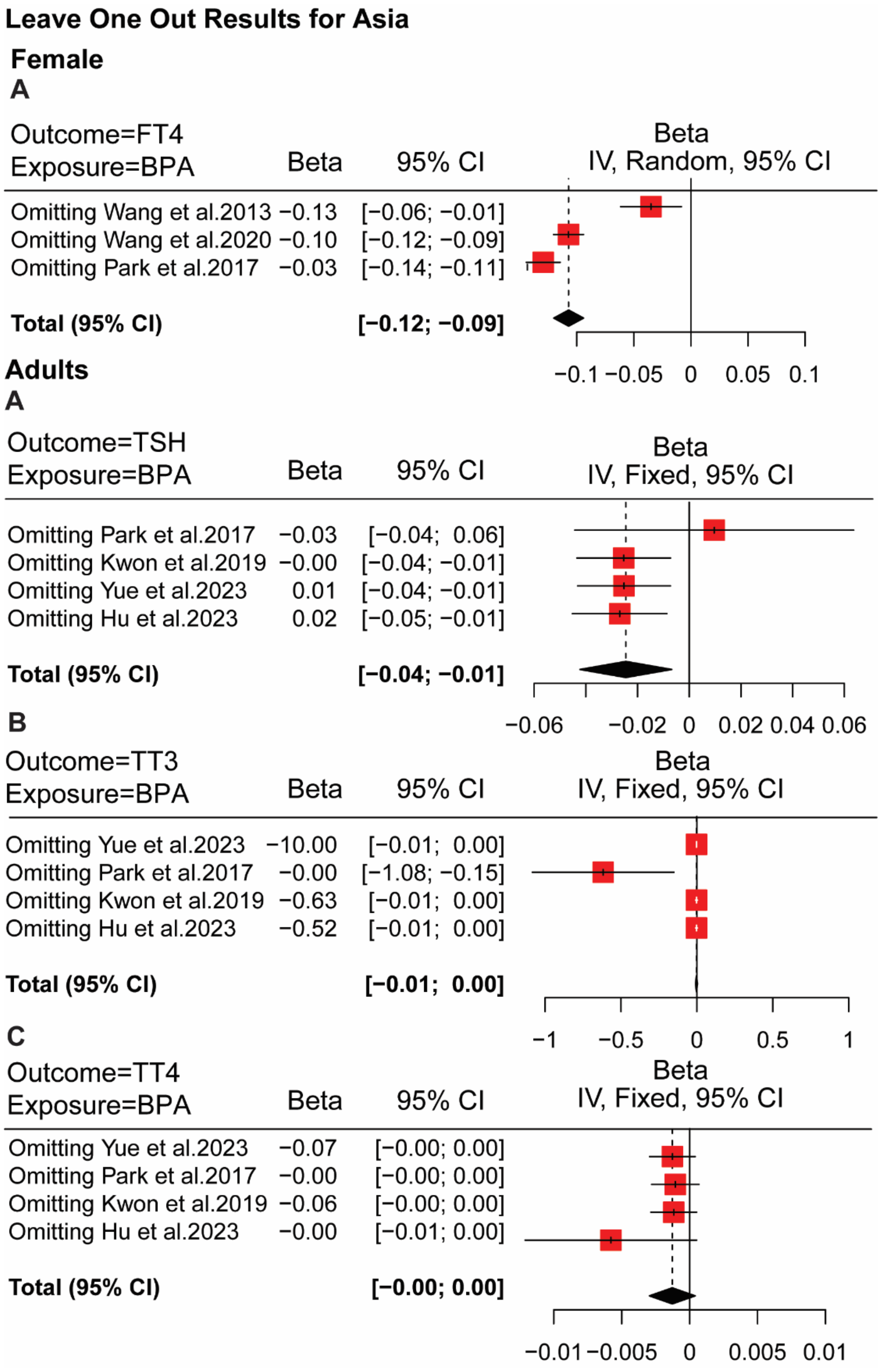

Figure S20: leave one out method results for Asia region

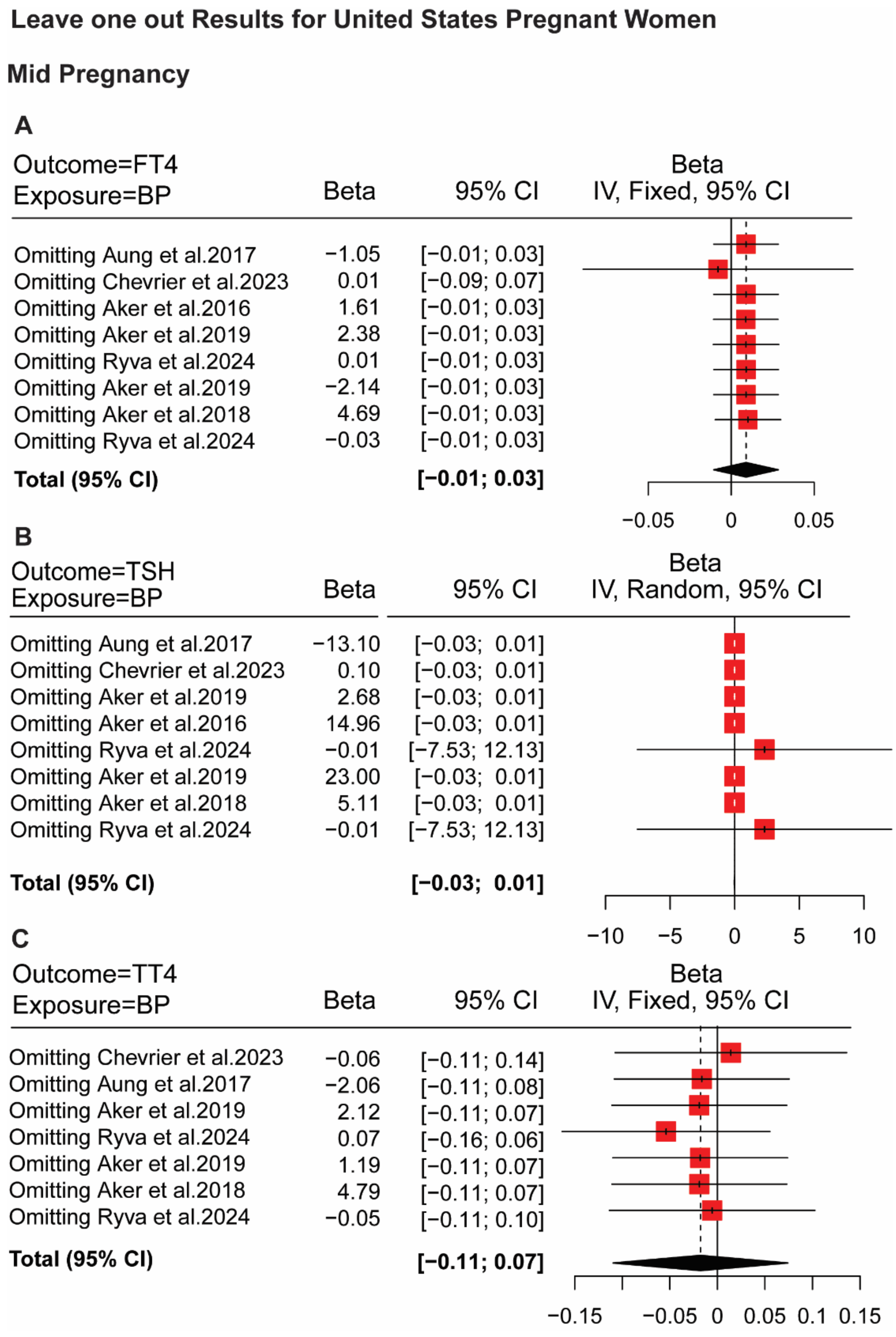

Figure S21: leave one out method results for US region
